# TFAM promotes mitochondrial division by increasing mitochondrial Sirt3

**DOI:** 10.1038/s41419-026-08750-w

**Published:** 2026-04-21

**Authors:** Shixian Zhai, Zihong Huang, Zewei Luo, Chunchun An, Lu Gao, Tongsheng Chen

**Affiliations:** MOE Key Laboratory of Laser Life Science & Guangdong Provincial Key Laboratory of Laser Life Science, College of Biophotonics, South China Normal University, Guangzhou, China

**Keywords:** Mitochondria, Enzyme mechanisms

## Abstract

Mitochondrial transcription factor A (TFAM) plays a crucial role in mitochondrial fission beyond its canonical function in mtDNA maintenance. However, how TFAM regulates mitochondrial fission remains only partially understood. Fluorescence microscopy and TEM analyses showed that TFAM knockdown inhibited mitochondrial fission, whereas TFAM overexpression promoted mitochondrial fragmentation, and this mitochondrial morphology phenotype was supported by TEM-based ultrastructural observations in zebrafish embryos with *tfam* disruption. Depletion of Drp1 and MFF in TFAM-overexpressing cells led to elongated mitochondria, indicating that TFAM promotes Drp1- and MFF-dependent mitochondrial fission, which was further supported by the inhibitory effects of Mdivi-1 (Drp1 inhibitor) and Compound C (AMPK inhibitor) on TFAM-induced mitochondrial fission. Western blot and immunofluorescence analyses revealed that TFAM overexpression enhanced the mitochondrial localization and Sirt3-dependent mitochondrial protein deacetylation of Sirtuin 3 (Sirt3), increased phosphorylation of AMPK and MFF, and promoted mitochondrial recruitment of phosphorylated Drp1. Proteinase K protection and cycloheximide chase assays further supported intramitochondrial localization of Sirt3 and increased stability of mitochondrial Sirt3 upon TFAM overexpression. FRET imaging and co-immunoprecipitation demonstrated a direct TFAM-Sirt3 interaction mediated by TFAM’s HMG-box A domain. Targeted mutagenesis or deletion of the HMG-box A domain disrupted the TFAM-Sirt3 interaction, impaired Sirt3 mitochondrial localization and Sirt3-dependent mitochondrial protein deacetylation, and abolished TFAM-mediated mitochondrial fission. Analysis of TCGA data showed that high TFAM-SIRT3 co-expression is associated with overall survival across cancers, particularly in Kidney Renal Clear Cell Carcinoma (KIRC), where TFAM is downregulated (whereas SIRT3 is not). Together, these findings demonstrate that TFAM promotes mitochondrial fission via direct interaction with Sirt3, thereby activating the AMPK/MFF/Drp1 pathway.

## Introduction

Mitochondrial dynamics, which involve a balance between fusion and fission, represent a critical quality control mechanism essential for cellular homeostasis [1]. The fission process segregates damaged mitochondrial components for mitophagic degradation, while fusion allows for content mixing to buffer functional deficits [2, 3]. Imbalances in mitochondrial fusion and fission are hallmarks of numerous pathologies, including neurodegenerative conditions like Parkinson’s and Alzheimer’s diseases, metabolic syndromes, and cancer, highlighting the therapeutic potential of targeting mitochondrial dynamics regulators [2, 3]. Mitochondrial fission is executed by the cytosolic GTPase dynamin-related protein 1 (Drp1), which is recruited to the mitochondrial outer membrane primarily by the mitochondrial fission factor (MFF) [4]. While the core components of the fission machinery are well-defined, the upstream signaling events that spatially and temporally control the fission process remain incompletely understood.

Recent evidence has linked mitochondrial transcription factor A (TFAM), a key regulator of mitochondrial DNA (mtDNA) packaging, to the regulation of mitochondrial division [5]. TFAM-marked nucleoids preferentially localize to endoplasmic reticulum (ER)-mitochondria contact sites, which are recognized as hotspots for mitochondrial fission events [6, 7]. Within such contact sites, TFAM serves as a docking platform for motor proteins to orchestrate nucleoid transport [6]. In support of a regulatory role for TFAM, bezafibrate—a compound known to upregulate TFAM expression [8]—has been reported to restore mitochondrial fission efficiency and improve ultrastructure in Drp1-deficient cells [9]. The combined spatial and functional evidence positions TFAM as a regulatory component. However, a significant knowledge gap persists: the molecular mechanism by which TFAM’s presence at fission sites influences the core fission machinery, such as Drp1 and MFF, remains to be elucidated.

Sirtuin 3 (Sirt3), a primary mitochondrial NAD⁺-dependent deacetylase [10]. has emerged as a candidate to connect TFAM with the fission machinery. Sirt3 is known to physically interact with TFAM [11]. Separately, Sirt3 activates AMP-activated protein kinase (AMPK) [10, 12], a kinase that promotes mitochondrial fission via phosphorylation of MFF, thereby facilitating Drp1 recruitment [3]. Although prior studies suggest Sirt3 is involved in both TFAM regulation and mitochondrial fission (potentially as an upstream regulator of AMPK), a key question remains: does TFAM recruit Sirt3 to drive the AMPK-dependent fission pathway, thereby linking TFAM’s spatial localization at fission sites with the downstream fission machinery? We hypothesized that TFAM promotes mitochondrial fission by directly binding Sirt3, thereby augmenting Sirt3-dependent deacetylation in mitochondria and activating the AMPK/MFF/Drp1 pathway.

Here, we investigated the molecular mechanism linking TFAM to mitochondrial fission and assessed clinical associations using public transcriptomic datasets. We demonstrate that TFAM directly interacts with Sirt3 via the High Mobility Group-box A (HMG-box A) domain of TFAM. The TFAM-Sirt3 interaction facilitates the mitochondrial localization of Sirt3 and enhances Sirt3-dependent mitochondrial protein deacetylation (mito Ac-K), leading to activation of the AMPK/MFF/Drp1 mitochondrial fission pathway. Bioinformatic analysis of The Cancer Genome Atlas (TCGA) further supports the clinical association of TFAM-SIRT3 co-expression, showing that TFAM is selectively downregulated in Kidney Renal Clear Cell Carcinoma (KIRC) and that high TFAM-SIRT3 co-expression is associated with overall survival in multiple cancer types. Our findings describe a non-canonical role for TFAM and elucidate the TFAM-Sirt3-AMPK/MFF/Drp1 pathway in mitochondrial fission, providing a framework for future studies of its disease relevance.

## Materials and methods

### Reagents, antibodies and constructs

Key cell culture media, pharmacological reagents, dyes, commercial kits, and all primary and secondary antibodies are listed in Supplementary Tables S1 and S2 with suppliers and catalogue numbers. Plasmids and viral vectors (expression constructs, shRNA vectors and epiCRISPR-based knockout constructs), including TFAM, MFF and SIRT3 fusion and mutant plasmids, as well as guide-RNA oligonucleotides and primers, are summarized in Supplementary Table S3. Software packages and web-based tools used in this study are listed in Supplementary Table S4. The mitochondria-targeted mCherry-ActA construct was provided by David W. Andrews [13], and the epiCRISPR backbone vector was provided by Dr. Wang [14]. All other constructs were generated by standard PCR-based subcloning and site-directed mutagenesis, as detailed in Supplementary Table S3.

### Cell lines and cell culture

MCF-7, U2OS, HeLa, HEK293T, COS7 and IOSE-80 cells were obtained from the Cell Bank of the Chinese Academy of Medical Sciences. MCF-7 cells were cultured in DMEM and U2OS cells in McCoy’s 5 A medium, both supplemented with 10% FBS and 1% penicillin–streptomycin. Unless otherwise stated, HeLa, HEK293T, COS7 and IOSE-80 cells were cultured in their recommended basal media with 10% FBS and 1% penicillin–streptomycin. All cell lines were maintained at 37 °C in a humidified incubator with 5% CO₂ and used in the logarithmic growth phase. For each experiment, all treatment groups were seeded from the same passage number and culture flask and processed in parallel. All cell lines were recently authenticated by short tandem repeat (STR) profiling and were routinely tested and confirmed to be free of mycoplasma contamination. No formal randomization procedure was performed.

### Cell transfection and lentiviral infection

For transient transfections, cells at ~60% confluency were transfected using a DNA-to-reagent ratio of 1 μg:2 μL. The complexes were formed in serum-free DMEM for 15 min before being added to cells, which were then cultured for at least 12 h prior to analysis. For lentivirus production, 293T cells were co-transfected via the calcium phosphate method with a shRNA expression vector (targeting TFAM, Sirt3, or control) and packaging plasmids (pPAX2, pMD2.G). Viral supernatant was collected after 48 h, filtered (0.45 µm), and used to transduce target cells overnight in the presence of 8 µg/mL Polybrene. Stably expressing cells were selected using 2 µg/mL puromycin.

### Zebrafish *tfam* knockdown and validation

Wild-type zebrafish (*Danio rerio*, AB strain) were maintained at 28.5 °C on a 14 h light/10 h dark cycle under standard conditions. Embryos of either sex were used; sex was not determined at embryonic stages. An sgRNA targeting zebrafish *tfam* (TATGCTGTCAGGGGTCTCTT) was diluted in Danieau’s solution containing 0.1% phenol red, and approximately 1–2 nL was microinjected into one-cell-stage embryos according to the manufacturer’s instructions. Knockdown efficiency was assessed at 24 hpf by PCR amplification and deep sequencing of the *tfam* locus, which revealed a total cutting efficiency of 83.3%. At 5 dpf, embryos were processed for TEM-based ultrastructural analysis of mitochondria in somitic muscle cells. Embryos from the same clutches were allocated to control and *tfam* sgRNA groups and processed in parallel; no formal randomization procedure was applied.

### Western blot and co-immunoprecipitation (Co-IP)

Proteins were resolved by 10% SDS-PAGE, transferred to PVDF membranes, and blocked with 5% BSA. Membranes were incubated with primary antibodies (1:1000) overnight at 4 °C, followed by HRP-conjugated secondary antibodies and ECL detection. Band intensities were quantified using ImageJ. For the proteinase K (PK) protection assay, isolated mitochondria were left untreated (No PK), treated with PK in the absence of detergent, or treated with PK in the presence of Triton X-100, followed by immunoblotting for Sirt3 and mitochondrial marker proteins to assess protease protection. For cycloheximide (CHX) chase experiments, cells were treated with CHX and mitochondrial fractions were collected at the indicated time points for immunoblot analysis of mitochondrial Sirt3.

For Co-IP, cells transfected with YFP-Sirt3 and/or TFAM-MYC plasmids were lysed after 24–48 h. Lysates were immunoprecipitated overnight with anti-GFP antibody (2 μg), and immunocomplexes were captured with Protein A/G beads. After washing, bound proteins were eluted and analyzed by Western blot for TFAM-MYC (anti-MYC) and YFP-Sirt3 (anti-GFP), alongside a 5% input control. Full-length, uncropped Western blot images corresponding to the figures in the main text and supplementary figures are provided in the supplementary file “Uncropped_Blots”.

### Quantification of mitochondrial morphology

For quantification of mitochondrial morphology in MitoTracker-stained cells, scoring was performed in a blinded manner with respect to genotype and treatment. Mitochondria were categorized as “fragmented” (predominantly spherical), “short” (majority < 8 μm) or **“**long**”** (majority > 8 μm). Colocalization was quantified by particle analysis in ImageJ and normalized to the total mitochondrial area in each image. For quantitative analysis of aspect ratio, elongation and interconnectivity, reconstructed structured illumination microscopy (SIM) images were processed and binarized in ImageJ, followed by particle analysis to obtain mitochondrial morphology parameters per cell. Aspect ratio was used as an index of mitochondrial shape, mitochondrial elongation was measured by inverse circularity, and mitochondrial interconnectivity by the mean area-to-perimeter ratio, according to published protocols [15, 16]. For transmission electron microscopy (TEM; Hitachi HT7800, Tokyo, Japan), mitochondrial profiles were manually traced in ImageJ from randomly selected fields, and mitochondrial length and aspect ratio were calculated from the fitted ellipse for each mitochondrion. TEM images were coded and analyzed under blind conditions with respect to genotype and treatment.

### Mitochondrial and cytosolic fractionation

Mitochondrial and cytosolic fractions were prepared using a commercial kit, and protein content was quantified via BCA assay. Equal protein amounts (20 µg) per fraction were subjected to Western blot analysis. Samples were resolved by SDS-PAGE, transferred to PVDF membranes, and probed with relevant primary antibodies. Blots were developed with HRP-conjugated secondary antibodies and ECL reagent.

### Immunofluorescence

Cells on coverslips were sequentially fixed (4% PFA), permeabilized (0.2% Triton X-100), and blocked (5% BSA). Samples were then incubated with relevant primary antibodies overnight at 4 °C, followed by a 1-h incubation with Alexa Fluor-conjugated secondary antibodies (1:2000) and DAPI (1:2000) at room temperature. Images of mounted samples were acquired on an Olympus IX73 (Tokyo, Japan) inverted fluorescence microscope.

### Live-cell fluorescence resonance energy transfer (FRET) assay

FRET is an imaging technique that allows for in situ real-time monitoring of a weakly reversible dynamic molecular event in a living cell [17–19]. FRET imaging was performed using a Wide-field Fluorescence Multi-modal FRET Imaging System as previously described [20, 21]. Donor-centric FRET efficiency (*E*_*D*_) and acceptor–donor ratio (*Rc*) were measured using the FRET method as previously described, and the saturation curve was fitted using Origin software with the function: *E*_*D*_ *= E*_*Dmax*_ *× Rc/ (K*_*d*_ + *Rc)* [18, 22–24]. Live-cell FRET experiments provide a means to monitor protein interactions in real time within living cells [17–19].

### Protein–protein docking

Protein-protein docking was performed using the HDOCK program [25–27]. which utilizes a hybrid docking strategy [27]. HDOCK is freely available for academic use. A total of 10 binding poses were generated for the protein complex. These poses were scored using the integrated ITScorePP knowledge-based iterative scoring function [28, 29]. The conformation exhibiting the lowest docking score (where a more negative score indicates a more probable binding model) was selected as the final predicted complex structure. Subsequent analysis and visualization of the resulting protein-protein complex model were carried out using PyMOL software.

### Bioinformatic analysis of public datasets

De-identified gene expression and clinical data from TCGA and GTEx were analyzed using the GEPIA2 and GEPIA3 web servers. Differential expression of TFAM and SIRT3 in KIRC was assessed in GEPIA3 [30] by comparing TCGA tumor samples with a combined TCGA/GTEx normal cohort using Student’s *t* test. All other expression and correlation analyses were performed in GEPIA2 [31] with Pearson correlation used to evaluate the relationship between TFAM and SIRT3. For survival analysis, patients were stratified according to a combined two-gene TFAM-SIRT3 expression signature, and overall survival (OS) was analyzed using Kaplan–Meier curves, the log-rank test and hazard ratios (HR) for the high-expression group. *P* values < 0.05 were considered statistically significant.

### Statistical analysis

Data are expressed as mean ± standard deviation (SD) from at least three independent experiments, unless otherwise indicated. All statistical analyses were performed using GraphPad Prism version 10.0. Comparisons between two groups were conducted using a two-tailed Student’s *t* test, whereas one-way analysis of variance (ANOVA) followed by Tukey’s post hoc test was used for multiple-group comparisons. Statistical significance was defined as follows: ns, not significant (*P* ≥ 0.05); *P < 0.05; **P < 0.01; ***P < 0.001; and ****P < 0.0001. No formal priori sample size calculation was performed; sample sizes for cell and zebrafish experiments were based on previous studies and our experience with similar assays and were considered sufficient to detect the expected differences in mitochondrial phenotypes. No samples, animals or data points were excluded except in cases of predefined technical failure (e.g., loss of staining or obvious imaging artifacts). Formal tests for normality or homogeneity of variance were not performed; however, data distributions appeared approximately symmetric and group variances were similar upon visual inspection. Except for the blinded analyses of mitochondrial morphology and TEM images described above, investigators were not blinded to group allocation during data collection and analysis.

## Results

### TFAM plays a key role in mitochondrial fission

**Fig. 1:**
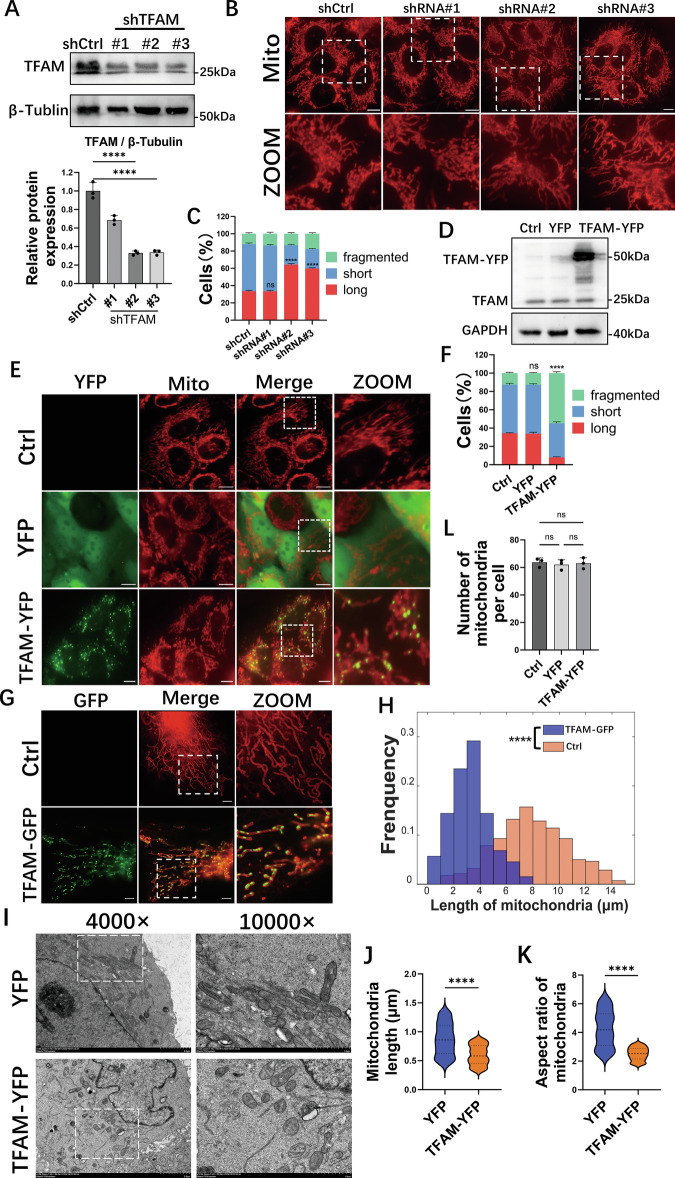
TFAM regulates mitochondrial fission. **A** Western blot of TFAM in MCF-7 cells expressing shCtrl or TFAM shRNAs. Live-cell images of mitochondria labeled with MitoTracker Deep Red in TFAM-depleted (**B**) or TFAM–YFP-overexpressing (**E**) cells. Scale bars, 10 μm. **C**, **F**, **L** Quantification of mitochondrial morphology and number per cell from (**B**, **E**). **D** Western blot validation of TFAM–YFP overexpression. **G** SIM images of mitochondria labeled with mCherry–ActA in U2OS cells with or without TFAM–GFP. Scale bar, 5 μm. **H**–**K** Quantification of mitochondrial length and aspect ratio from SIM images (**G**) and TEM images (**I**) in YFP and TFAM–YFP cells. **I** TEM images of mitochondrial morphology. Scale bars, 2 μm and 1 μm. Violin plots show all mitochondria. For **C**, **F**, **L**, *n* = 100 cells per condition in each of three independent experiments. For **H**, **J**, **K**, *n* = 180 mitochondria from 60 cells per condition, pooled from three experiments. Data are mean ± SD. One-way ANOVA was used for (**C**, **F**) and Student’s *t* test for other quantifications. ****P < 0.0001, **P < 0.01, ns, not significant.

To investigate the role of TFAM in mitochondrial dynamics, TFAM was depleted in MCF-7 cells using three distinct shRNAs. TFAM protein reduction by approximately 70% and 65% was confirmed by Western blot for shRNA-2 and shRNA-3, respectively, compared to controls (Fig. 1A). Following live-cell imaging of mitochondria, cells were categorized for quantitative analysis based on mitochondrial morphology as “Long”, “Short”, or “Fragmented” (Fig. 1B). A decrease in the percentage of cells with fragmented mitochondria was observed in the shRNA-2 and shRNA-3 groups, accompanied by an increase in the percentage of cells with elongated mitochondrial structures (Fig. 1C). These results suggest that TFAM knockdown suppresses mitochondrial fission, leading to an elongated mitochondrial network. Conversely, a TFAM-YFP fusion protein was overexpressed in MCF-7 cells, confirmed by Western blot (Fig. 1D). Overexpression of TFAM-YFP significantly increased the proportion of cells with fragmented mitochondria (Fig. 1E, F). Overexpression of TFAM-YFP in HeLa, COS-7 and IOSE-80 cells also increased the proportion of cells with fragmented mitochondria (Fig. S1A), indicating that TFAM promotes mitochondrial fragmentation in both cancer cells and non-malignant cells.

To quantify the morphological changes at high resolution, we used a custom-built Structured Illumination Microscopy (SIM) system [32]. to image U2OS cells co-expressing TFAM-GFP and a mitochondrial marker (Fig. 1G, H). In cells overexpressing TFAM-GFP, mitochondria were observed to be significantly shorter (Fig. 1G, H). Further quantitative analysis confirmed a significant reduction in mitochondrial aspect ratio, network interconnectivity, and elongation, indicative of a fragmented mitochondrial network (Fig. S1B–D). To further validate the morphological changes at the ultrastructural level, transmission electron microscopy (TEM) was employed. TEM analysis revealed that, compared to the elongated tubular mitochondria in control cells, mitochondria in cells overexpressing TFAM-YFP were mainly fragmented into smaller, ovoid structures (Fig. 1I). Quantitative morphometric analysis confirmed significant decreases in mitochondrial length and aspect ratio in cells overexpressing TFAM-YFP (Fig. 1J, K). These observations provide ultrastructural evidence consistent with TFAM promoting mitochondrial fission and a fragmented mitochondrial morphology.

To determine whether TFAM-mediated changes in mitochondrial morphology reflected altered mitochondrial health or increased biogenesis, we assessed mitochondrial membrane potential, ROS levels, total mitochondrial mass, and mtDNA copy number (Figs. 1L and S2A–D). These analyses indicate that TFAM bidirectionally regulates mitochondrial morphology by promoting mitochondrial fission without inducing mitochondrial stress or enhanced biogenesis.

### In vivo TEM analysis of mitochondrial morphology following *tfam* disruption in zebrafish

**Fig. 2:**
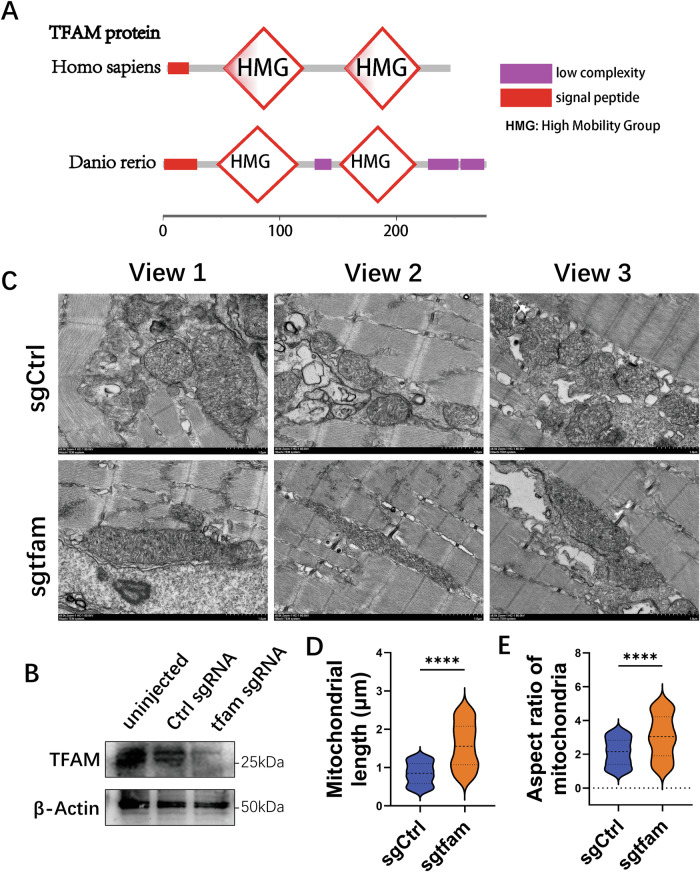
TFAM conservation analysis and mitochondrial morphology in zebrafish. **A** Domain organization of human and zebrafish TFAM. **B** Western blot of TFAM in uninjected, control sgRNA-injected and *tfam* sgRNA-injected embryos. **C** TEM images of somitic muscle mitochondria in sgCtrl and sg*tfam* embryos at 5 dpf. Scale bars, 1 μm. Quantification of mitochondrial length (**D**) and aspect ratio (**E**). Violin plots show median and quartiles; each point is one mitochondrion (*n* = 50 mitochondria from 20 embryos per condition). Data are representative of three independent experiments. ****P < 0.0001 (Student’s *t* test).

To evaluate whether the cell-based mitochondrial morphology phenotype can also be observed in vivo at the ultrastructural level, we used a zebrafish (Danio rerio) model. Comparative analyses revealed high sequence similarity between zebrafish and human TFAM (68% similarity; E-value = 7 × 10⁻⁵⁴), and the HMG-box A/B domains showed particularly high conservation, supporting the use of zebrafish as a comparative model to examine TFAM-associated mitochondrial phenotypes (Fig. 2A; gene organization and full-length alignments are provided in Supplementary Fig. S3A, B). Endogenous *tfam* disruption was induced by injection of a *tfam*-targeting sgRNA in zebrafish embryos, with disruption efficiency confirmed by sequencing and Western blot (Figs. S3C and 2B). Embryos at 5 days post-fertilization (dpf) were embedded in resin, and ultrathin sections of somitic muscle tissue were prepared for TEM-based ultrastructural analysis of mitochondria. TEM analysis revealed that *tfam*-disrupted embryos exhibited mitochondrial elongation and hyperfusion compared to controls (Fig. 2C). Quantitative analysis confirmed significant increases in mitochondrial length and aspect ratio upon *tfam* disruption (Fig. 2D, E). Together, these results provide in vivo ultrastructural evidence that *tfam* disruption is associated with mitochondrial elongation and hyperfusion, consistent with our cell-based observations.

### AMPK/MFF/Drp1 play key roles in TFAM-mediated mitochondrial fission

**Fig. 3:**
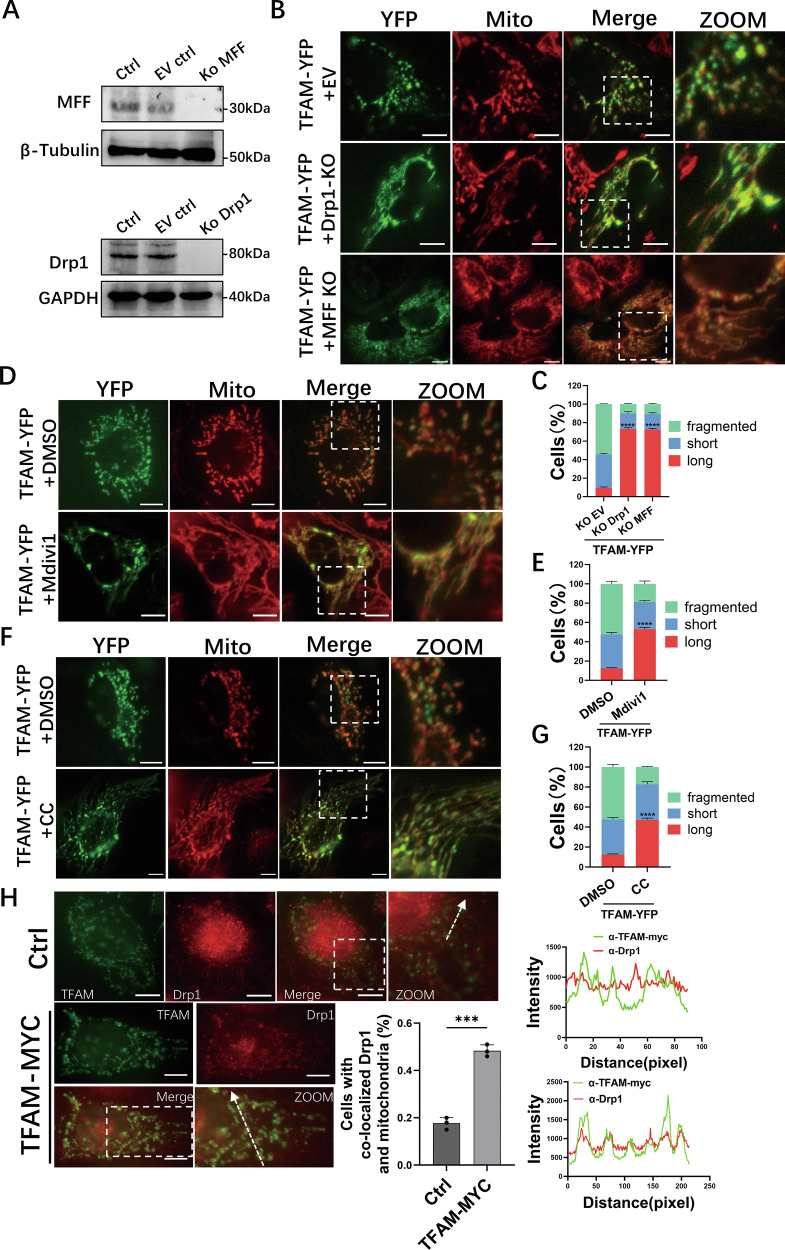
The AMPK/MFF/DRP1 pathway is involved in TFAM-mediated mitochondrial fission. **A** Western blot validation of Drp1 and MFF knockout in MCF-7 cells. **B** MitoTracker-labeled mitochondria in EV (transfected with the empty knockout vector), Drp1-KO and MFF-KO cells expressing TFAM–YFP. **C** Quantification of mitochondrial morphology from (**B**). **D** Mitochondria in TFAM–YFP cells treated with DMSO or Mdivi-1 (10 μM, 6 h). **E** Quantification from (**D**). **F** Mitochondria in TFAM–YFP cells treated with DMSO or CC (10 μM, 12 h). **G** Quantification from (**F**). **H** Drp1 (red) and TFAM–MYC (green) in control and TFAM–MYC cells, with insets, co-localization analysis and intensity line scans. For **C**, **E**, **G**, *n* = 80 cells per condition; for (**H**), *n* = 60 cells per condition. Data are mean ± SD from three independent experiments. One-way ANOVA was used for (**C**) and Student’s *t* test for (**E**, **G**, **H**). ****P < 0.0001, ***P < 0.001. Scale bars, 10 μm.

To investigate the involvement of the AMPK/MFF/Drp1 pathway in TFAM-mediated fission, Drp1 and MFF were independently knocked out (KO) in MCF-7 cells using CRISPR-Cas9 (Fig. 3A). As a baseline, depletion of either protein in control cells transfected with the empty vector (EV) expressing YFP resulted in elongated mitochondria, as assessed by live-cell fluorescence imaging (Fig. S4A, B), providing a reference for mitochondrial morphology in the absence of Drp1 or MFF. Overexpression of TFAM-YFP significantly promoted mitochondrial fragmentation in control (EV) cells, whereas fragmentation was not increased in Drp1-KO or MFF-KO cells (Figs. 3B, C and S4A, B). These findings indicate that TFAM-mediated fission requires both MFF and Drp1.

The requirement for Drp1 and AMPK activity was further confirmed pharmacologically. In cells overexpressing TFAM, treatment with the Drp1 inhibitor Mdivi-1 suppressed mitochondrial fragmentation, restoring an elongated phenotype as assessed by live-cell fluorescence imaging (Fig. 3D, E). Similarly, treatment with the AMPK inhibitor Compound C (CC) prevented TFAM-mediated fragmentation and led to a significant increase in the proportion of cells with elongated mitochondria (Fig. 3F, G). To gain mechanistic insight, immunofluorescence imaging was performed on cells overexpressing TFAM. A clear translocation of Drp1 (labeled red) to mitochondria was observed (Fig. 3H), providing evidence of Drp1 recruitment to mitochondria following TFAM overexpression. Collectively, the genetic, pharmacological, and imaging data indicate that TFAM-mediated mitochondrial fission is dependent on the AMPK/MFF/Drp1 pathway.

### TFAM regulates the AMPK/MFF/Drp1 pathway to regulate mitochondrial fission

**Fig. 4:**
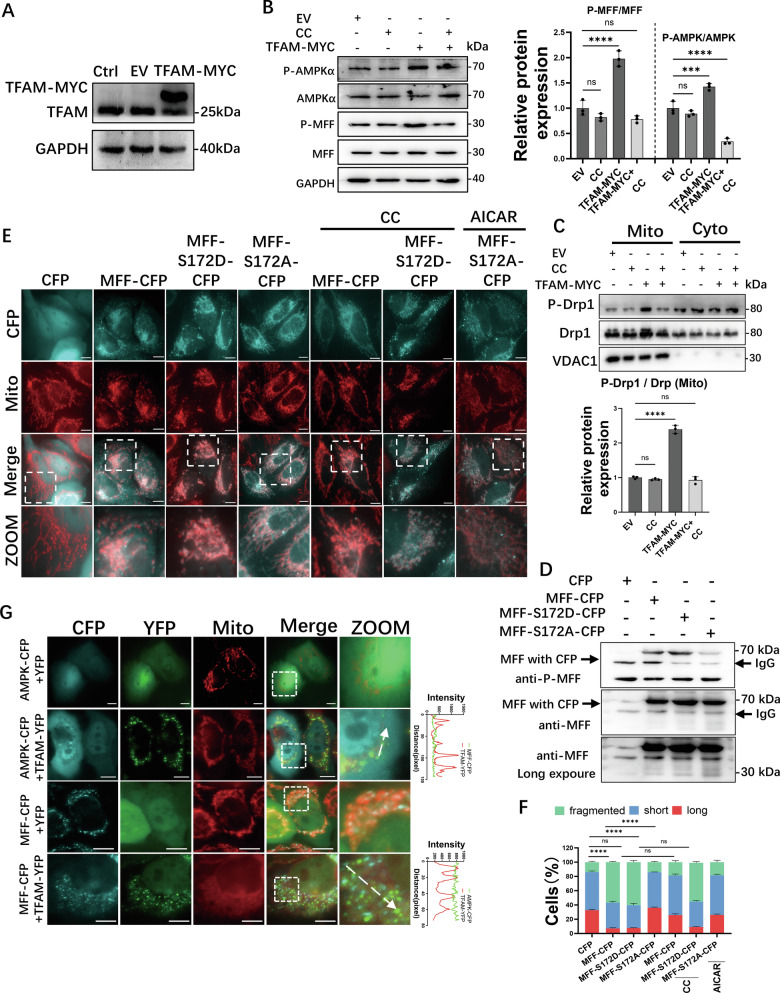
TFAM regulates mitochondrial fission via AMPK/MFF phosphorylation. **A** Western blot validation of TFAM–MYC overexpression in MCF-7 cells. **B** Phosphorylated AMPKα (P-AMPKα) and MFF (P-MFF) in EV or TFAM–MYC cells with or without CC (10 μM, 12 h). **C** Cytoplasmic and mitochondrial fractions from EV or TFAM–MYC cells ± CC (20 μM, 6 h). **D** Phospho-MFF and total MFF in cells expressing CFP, MFF–CFP, MFF–S172D–CFP or MFF–S172A–CFP; longer exposure of anti-MFF is shown. **E** MitoTracker-labeled mitochondria in cells expressing CFP, MFF–CFP, or the phospho-mutants with or without CC (10 μM, 12 h) or AICAR (500 μM, 12 h). **F** Quantification of mitochondrial morphology from (**E**); *n* = 80 cells per condition. **G** Co-localization analysis of AMPK–CFP or MFF–CFP with YFP or TFAM–YFP, with MitoTracker and line scans. Data in **B**, **F** are mean ± SD from three independent experiments. One-way ANOVA was used for statistical analysis. ****P < 0.0001, ***P < 0.001, ns, not significant. Scale bars, 10 μm.

To investigate the regulatory role of TFAM in mitochondrial dynamics, MCF-7 cells stably overexpressing TFAM-MYC were generated using a lentiviral system. Western blot confirmed significant overexpression of TFAM compared to the controls (EV) (Fig. 4A). Notably, cells overexpressing TFAM exhibited significantly increased phosphorylation of both AMPK (P-AMPK) and the AMPK downstream target MFF (P-MFF), and also showed accumulation of Drp1 and phospho-Drp1 (P-Drp1 S616) specifically within the mitochondrial fraction (Fig. 4B, C). We treated cells with CC to determine whether the recruitment and activation of Drp1 were dependent on AMPK signaling. CC treatment suppressed the phosphorylation of both AMPK and MFF, and consequently attenuated mitochondrial accumulation of Drp1 and phospho-Drp1 (Fig. 4B, C). These findings indicate that TFAM promotes the recruitment and subsequent activation of Drp1 at the mitochondria via an AMPK-MFF-dependent pathway, which was further supported by the observation that TFAM overexpression activated the AMPK/MFF/Drp1 pathway in HEK293T and HeLa cells (Fig. S5A–D).

Previous research has reported that the activation of AMPK significantly enhances MFF phosphorylation targeting the Serine 172 residues [3]. To further dissect the functional role of MFF phosphorylation in mitochondrial fission, phospho-mimetic (MFF-S172D-CFP; Serine 172 to Aspartic acid substitution, mimicking constitutive phosphorylation) and phospho-deficient (MFF-S172A-CFP; Serine 172 to Alanine substitution, abolishing phosphorylation potential) mutants fused to CFP were generated. Western blot analysis was performed using an antibody recognizing phosphorylation at both Ser172 and Ser146 of MFF (Fig. 4D). The wild-type MFF-CFP exhibited a clear phosphorylation signal, indicating phosphorylation at Ser172 and/or Ser146. The phospho-deficient MFF-S172A mutant exhibited near-complete loss of phosphorylation signal, confirming Ser172 as the primary phosphorylation site under basal conditions. Interestingly, the phospho-mimetic MFF-S172D mutant displayed a markedly enhanced phosphorylation signal compared to wild-type MFF. Since the antibody detects phosphorylation at both Ser172 and Ser146, and Ser172 is substituted with aspartate (which cannot be phosphorylated), the enhanced signal likely reflects increased phosphorylation at Ser146. This observation suggests that the S172D substitution may stabilize a specific MFF conformation that renders Ser146 more accessible to kinases. The impact of MFF phosphorylation on mitochondrial morphology was subsequently assessed. In cells overexpressing WT MFF-CFP, the proportion of cells with fragmented mitochondria increased, and CC treatment reversed mitochondrial fragmentation. Cells overexpressing the phospho-mimetic MFF-S172D-CFP mutant exhibited constitutive mitochondrial fragmentation, unaffected by CC (Fig. 4E, F). Conversely, the phospho-deficient MFF-S172A-CFP mutant maintained elongated mitochondria even under activation of AMPK by AICAR, an AMPK agonist (Fig. 4E, F). These results demonstrate that phosphorylated MFF is essential for mitochondrial fission and that TFAM regulates the mitochondrial fission process by enhancing AMPK/MFF phosphorylation. Notably, co-localization studies revealed no interaction between TFAM and either AMPK or MFF (Fig. 4G), suggesting an indirect regulatory mechanism. Together, these findings indicate that TFAM modulates the AMPK/MFF/Drp1 pathway and mitochondrial fission. However, a key question remains: how does TFAM influence AMPK/MFF/Drp1 signaling in the absence of detectable direct interactions with AMPK or MFF?

### TFAM increases mitochondrial Sirt3 to regulate mitochondrial fission

**Fig. 5:**
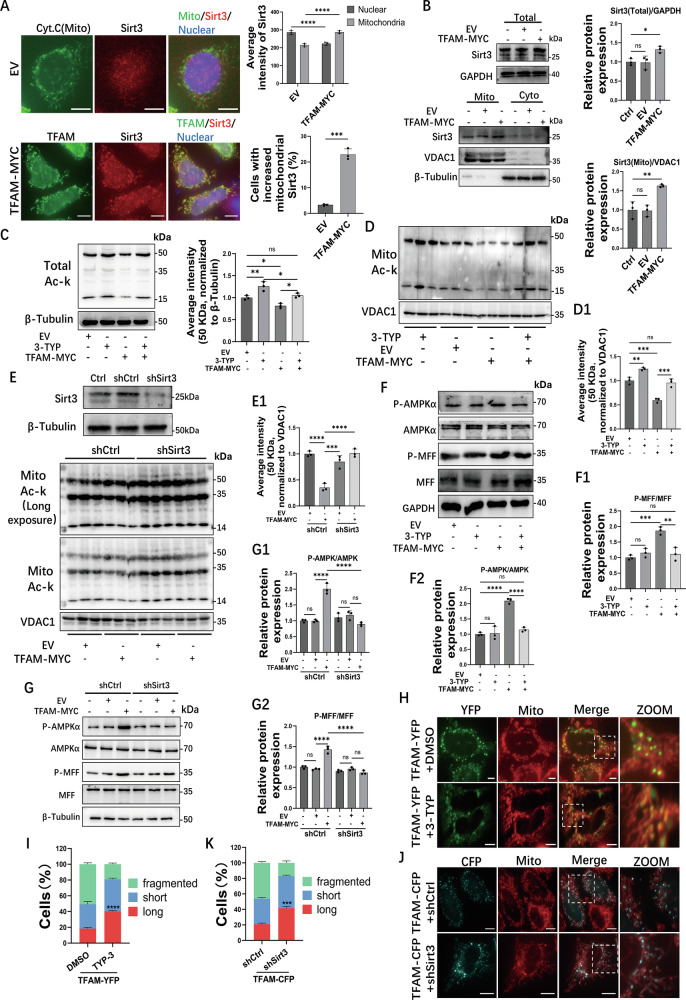
TFAM increases mitochondrial Sirt3 to regulate AMPK phosphorylation and mitochondrial fission. **A** Sirt3 (red) and mitochondria (cytochrome c, green) in EV or TFAM–MYC cells; nuclei, DAPI. Right, quantification of mitochondrial Sirt3 (*n* = 60 cells per condition). **B** Sirt3 in total, mitochondrial and cytosolic fractions. **C** Total protein acetylation (Ac-K) in EV or TFAM–MYC cells treated with DMSO or 3-TYP (10 μM, 12 h). **D** Mitochondrial Ac-K under the same conditions. **E** Sirt3 knockdown (shSirt3) and mitochondrial protein acetylation in TFAM–MYC cells. **F**, **G** AMPK and MFF phosphorylation in cells treated as in (**C**, **F**) or additionally expressing shCtrl or shSirt3 (**G**). **H** MitoTracker-labeled mitochondria in TFAM–YFP cells treated with DMSO or 3-TYP. **I** Quantification from (**H**). **J** Mitochondria in cells co-expressing TFAM–CFP with shCtrl or shSirt3. **K** Quantification from (**J**). For **I**, **K**, *n* = 80 cells per condition. Data are mean ± SD from three independent experiments. One-way ANOVA was used for (**B**–**G**) and Student’s *t* test for (**A**, **I**, **K**). ****P < 0.0001, ***P < 0.001, **P < 0.01, *P < 0.05, ns, not significant. Scale bars, 10 μm.

Based on reported interactions between TFAM and Sirt3 [11], we examined the effect of TFAM on Sirt3 to determine Sirt3’s role in regulating the AMPK/MFF/Drp1 pathway mediated by TFAM. TFAM overexpression in MCF-7 cells increased Sirt3 levels within mitochondria, as shown by immunofluorescence (Fig. 5A) and Western blot of mitochondrial fractions (Fig. 5B). TFAM overexpression in HEK293T cells also increased Sirt3 levels within mitochondria, as measured by Western blot (Fig. S6A). qPCR analysis showed no significant change in Sirt3 mRNA levels upon TFAM overexpression (Fig. S6B), indicating that TFAM regulates Sirt3 at a non-transcriptional level. Notably, while mitochondrial Sirt3 was enriched, total cellular Sirt3 exhibited only a modest increase (Figs. 5B and S6A). To further verify the sub-mitochondrial localization of Sirt3, we performed additional experiments in HEK293T cells. A proteinase K (PK) protection assay showed that Sirt3 remained protease-protected in isolated mitochondria, consistent with intramitochondrial localization of Sirt3 (Fig. S7A). Moreover, a cycloheximide chase assay indicated that TFAM overexpression increased the stability of mitochondrial Sirt3 (Fig. S7B). In MCF-7 cells, TFAM overexpression also enhanced Sirt3-dependent mitochondrial protein deacetylation, as evidenced by a decrease in protein acetylation detected by Western blot (Fig. 5C, D), which was reversed by either pharmacological inhibition with 3-TYP or genetic silencing of Sirt3 using shRNA (Fig. 5E). This conclusion was further supported by reduced acetylation of the canonical Sirt3 substrate SOD2 at lysine 68 (Ac-SOD2(K68)) upon TFAM overexpression in HEK293T cells, which was reversed by 3-TYP (Fig. S7C). Consistently, 3-TYP treatment also inhibited the TFAM-enhanced Sirt3-dependent mitochondrial protein deacetylation in HEK293T and IOSE-80 cells (Fig. S6C–F). Furthermore, 3-TYP treatment or silencing of Sirt3 attenuated TFAM-promoted phosphorylation of AMPK and MFF as well as Drp1 in MCF-7 cells (Fig. 5F, G), indicating that TFAM mediates Sirt3-dependent phosphorylation of AMPK and MFF as well as Drp1, which was also verified in HeLa, HEK293T, and IOSE-80 cells by 3-TYP treatment (Fig. S6G–L).

Finally, the functional consequence on mitochondrial morphology was assessed. While TFAM overexpression promoted mitochondrial fragmentation, pharmacological inhibition or silencing of Sirt3 led to a significant decrease in the proportion of cells with fragmented mitochondria induced by TFAM overexpression (Fig. 5H–K). Collectively, these findings show that TFAM enhances mitochondrial Sirt3 abundance and Sirt3-dependent mitochondrial protein deacetylation, which is essential for activating the AMPK/MFF/Drp1 pathway and promoting mitochondrial fission.

### TFAM’s HMG-box A domain is required for Sirt3 binding and Sirt3-dependent mitochondrial deacetylation

**Fig. 6:**
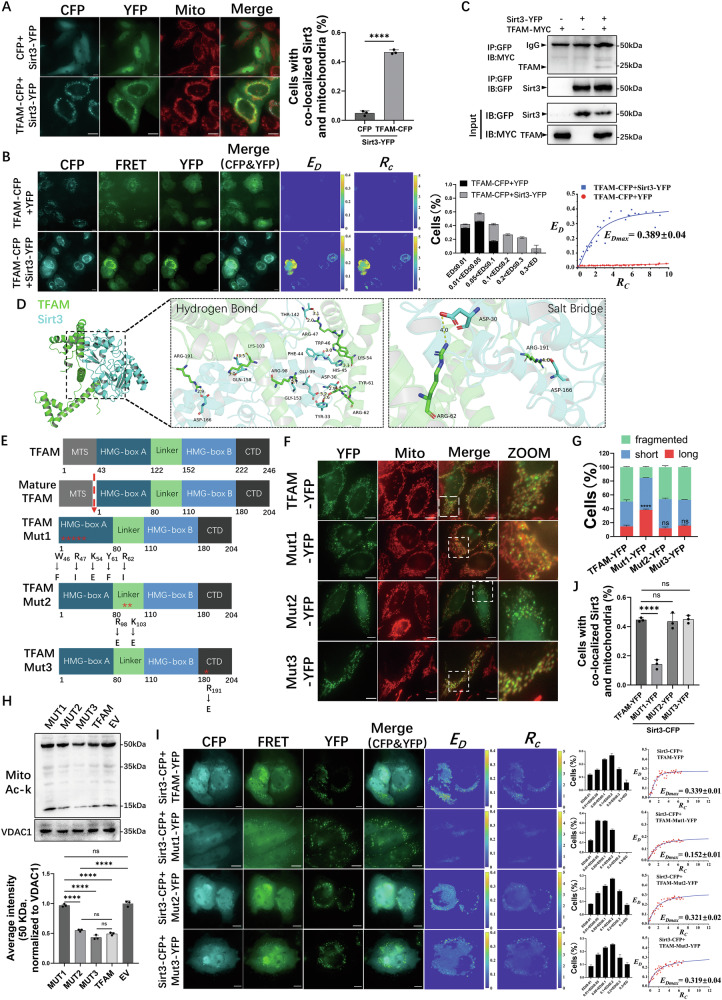
TFAM binds Sirt3 via HMG-box A to enhance intramitochondrial Sirt3 function. **A** Mitochondrial co-localization of Sirt3–YFP in cells co-expressing CFP or TFAM–CFP, with quantification. Mitochondria are labeled with MitoTracker. **B** Live-cell FRET between TFAM–CFP (donor) and Sirt3–YFP (acceptor), showing *E*_*D*_ and *Rc* maps, *E*_*D*_ distributions and *E*_*D*_–*Rc* plots. **C** Co-immunoprecipitation of Sirt3–YFP and TFAM–MYC. **D** Predicted TFAM (green)–Sirt3 (cyan) complex, with overall view and interface details highlighting hydrogen bonds and salt bridges. **E** Schematic of TFAM and mutations in HMG-box A (Mut1), linker (Mut2) and C-terminal (Mut3). **F** MitoTracker-labeled mitochondria in cells expressing WT TFAM–YFP or mutants. **G** Quantification of mitochondrial morphology from (**F**). **H** Total protein acetylation in cells expressing WT TFAM or mutants. **I** FRET between Sirt3–CFP and WT or mutant TFAM–YFP, with ED maps and plots. **J** Percentage of cells with mitochondrial Sirt3–YFP in TFAM or TFAM–Mut1/2/3 cells. For **A**, **B**, **G**, **I**, **J**, *n* = 80 cells per condition. Data are mean ± SD from three independent experiments. One-way ANOVA was used for (**G**, **J**) and Student’s *t* test for (**A**). ****P < 0.0001, ns, not significant. Scale bars, 10 μm.

To determine whether the regulation of Sirt3 by TFAM involves direct physical interaction, three approaches were used. Co-expression of TFAM-CFP and Sirt3-YFP in MCF-7 cells showed co-localization within mitochondria (Fig. 6A). Live-cell fluorescence resonance energy transfer (FRET) assays detected direct interaction, yielding a saturable FRET efficiency (*E*_*D*_, *E*_*D*max_ = 0.389 ± 0.04) for the TFAM-Sirt3 pair, which was absent in controls (Fig. 6B). This interaction was confirmed by co-immunoprecipitation, where Sirt3-YFP co-precipitated with TFAM-MYC from cell lysates (Fig. 6C).

To identify the binding interface, a structural model was generated. Based on Sirt3’s deacetylase activity [33] and the shielding of TFAM lysines upon DNA binding [34, 35]. The model predicted preferential interaction with non-DNA-bound, monomeric TFAM. Protein–protein docking of a 1:1 complex produced a highly favorable TFAM–Sirt3 model (score = −263.87 ), indicating a stable structure. Notably, the docking model highlighted a key interaction cluster within TFAM’s HMG-box A domain (Fig. 6D).

This prediction was tested using site-directed TFAM mutants: Mut1 (W46F, R47I, K54E, Y61F, R62I in HMG-box A), Mut2 (R98E, K103E in linker region), and Mut3 (R191E in C-terminal domain) (Fig. 6E). FRET analyses were performed to evaluate the interaction between TFAM mutants and Sirt3. As predicted, mutations in HMG-box A (TFAM-Mut1) markedly reduced the TFAM–Sirt3 interaction, evidenced by decreased FRET efficiency (Fig. 6I) and failure to increase mitochondrial enrichment of Sirt3-CFP as observed for wild-type TFAM (Fig. 6J). Consistently, in HEK293T cells, a proteinase K protection assay showed that TFAM-WT increased the amount of protease-protected (intramitochondrial) Sirt3, whereas TFAM-Mut1 failed to do so (Fig. S7A). Moreover, a cycloheximide chase assay indicated that TFAM-WT increased the stability of mitochondrial Sirt3, while TFAM-Mut1 largely lost this stabilizing effect (Fig. S7B). Loss of interaction was further validated by Western blot analysis, showing increased mitochondrial protein acetylation in cells overexpressing TFAM-Mut1 (Fig. 6H). In addition, immunoblotting of acetylated SOD2 at lysine 68 (Ac-SOD2 K68), a canonical Sirt3 target, showed that TFAM-WT reduced Ac-SOD2(K68) in HEK293T cells, whereas TFAM-Mut1 exhibited a markedly weaker effect (Fig. S7C). Ultimately, TFAM-Mut1 overexpression failed to promote mitochondrial fragmentation (Fig. 6F, G). In contrast, mutations in the linker (Mut2) or C-terminal domain (Mut3) did not affect the TFAM–Sirt3 interaction or TFAM-associated Sirt3-dependent mitochondrial protein deacetylation (Fig. 6H, I), nor did they affect mitochondrial fragmentation (Fig. 6F, G).

To further validate the role of the HMG-box A domain, a domain deletion approach was utilized. Two TFAM truncation mutants were generated: TFAM-ΔHMG-A (lacking the HMG-box A domain) and TFAM-ΔHMG-B (lacking the HMG-box B domain). Co-IP assays demonstrated that wild-type TFAM co-precipitated with Sirt3-CFP, whereas the TFAM-ΔHMG-A mutant exhibited significantly reduced interaction with Sirt3 (Fig. S8). In contrast, the TFAM-ΔHMG-B mutant retained binding to Sirt3 at levels similar to wild-type TFAM (Fig. S8).

Collectively, the data indicate that TFAM’s HMG-box A domain is required for the direct interaction between TFAM and Sirt3. The binding of TFAM to Sirt3 is a key upstream event for enhancing Sirt3 mitochondrial enrichment and Sirt3-dependent mitochondrial protein deacetylation, which in turn leads to the phosphorylation of the AMPK/MFF/Drp1 pathway and the execution of mitochondrial fission. These findings identify a TFAM-Sirt3 axis that controls mitochondrial fission, raising the question of whether this axis also influences tumor progression and patient prognosis.

### The TFAM-SIRT3 co-expression signature is associated with overall survival in cancer

**Fig. 7:**
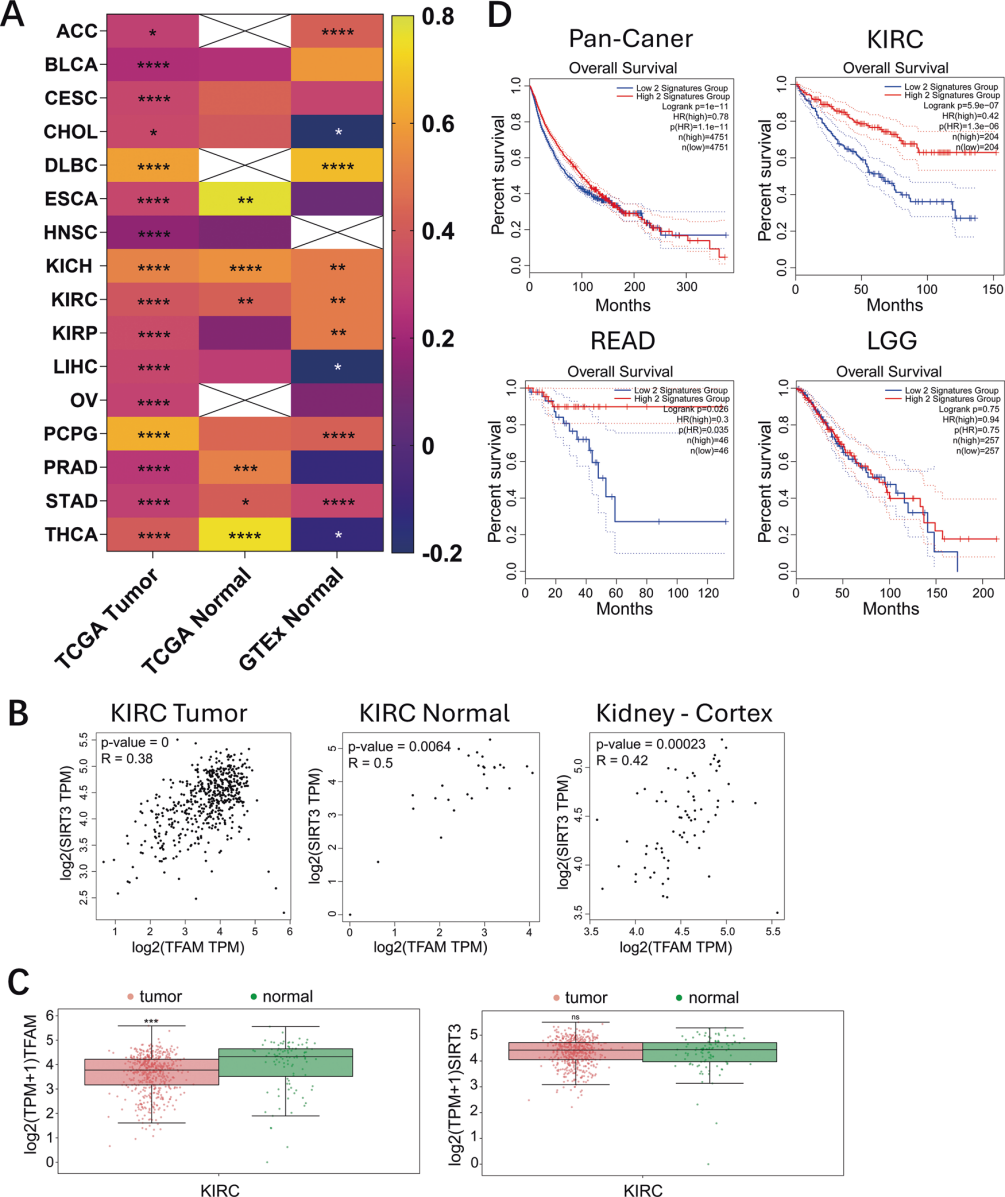
Co-expression of TFAM and SIRT3 is a favorable prognostic factor in multiple human cancers. **A** Heatmap of Pearson correlations between TFAM and SIRT3 mRNA across cancer types using TCGA and GTEx datasets. Colors show r; asterisks indicate significance (*P < 0.05, **P < 0.01, ***P < 0.001, ****P < 0.0001). **B** TFAM–SIRT3 correlations in KIRC tumors, KIRC normal-adjacent tissue and normal kidney cortex; r and P are shown. **C** TFAM and SIRT3 expression in KIRC. Boxplots compare log₂ (TPM + 1) in TCGA tumors (*n* = 531) versus TCGA + GTEx normal kidney (*n* = 100). t-test: ***P < 0.001; ns, not significant. **D** Kaplan–Meier curves for overall survival in patients stratified by TFAM–SIRT3 co-expression (High vs Low Signature Group) in pan-cancer, KIRC, READ and LGG. Log-rank P, hazard ratio (HR) for the high-expression group and group sizes (n) are indicated.

To assess the clinical associations of the TFAM-Sirt3-AMPK/MFF/Drp1 pathway, we analyzed TFAM and SIRT3 mRNA expression across multiple cancer types in TCGA. The analysis revealed significant positive correlations between TFAM and SIRT3 transcripts in numerous tumors, indicating a consistent co-expression pattern (Fig. 7A). A focused examination in kidney renal clear cell carcinoma (KIRC) confirmed the co-expression, with moderate positive correlations in tumor samples (*R* = 0.38, *p* < 0.001), normal-adjacent tissue (*R* = 0.50, *p* = 0.0064), and normal kidney tissue from GTEx (*R* = 0.42, *p* < 0.001; Fig. 7B). Despite the consistent positive correlation between TFAM and SIRT3 expression across samples, differential expression analysis revealed that only TFAM was significantly downregulated in KIRC tumors compared to non-malignant tissues (*p* < 0.001), whereas SIRT3 expression remained unchanged (*p* > 0.05, Fig. 7C). Based on the observed co-expression, we assessed the association of a combined TFAM-SIRT3 signature. Pan-cancer analysis of 9,502 patients showed that high signature scores were associated with better overall survival (OS) (HR = 0.78, 95% CI [0.74–0.83], log-rank *p* = 1.1 × 10⁻¹¹) (Fig. 7D). Individual tumor type analysis revealed context-dependent associations, notably with a strong effect in KIRC (HR = 0.42, 95% CI [0.31–0.57], log-rank *p* = 5.9 × 10^-7^) (Fig. 7D). Similar associations were observed in Rectum Adenocarcinoma (READ) (HR = 0.30, *p* = 0.035), but not in Low-Grade Glioma (LGG) (HR = 0.94, *p* = 0.75) (Fig. 7D).

Collectively, the data indicate an association between the TFAM-SIRT3 co-expression signature and overall survival, particularly in KIRC.

## Discussion

We identify a mechanism whereby TFAM promotes mitochondrial fission via direct interaction with Sirt3. TFAM enhances mitochondrial Sirt3 levels and Sirt3-dependent mitochondrial protein deacetylation, activating the AMPK/MFF/Drp1 pathway. Structural and functional analyses reveal TFAM’s HMG-box A domain as essential for Sirt3 binding, providing mechanistic insight into mitochondrial dynamics regulation.

Our observations that TFAM knockdown promoted mitochondrial network elongation, whereas TFAM overexpression promoted mitochondrial fragmentation (Fig. 1A–F) demonstrate that TFAM actively regulates mitochondrial dynamics, particularly the fission process. TFAM’s role in mtDNA maintenance is well-recognized [5]. The direct participation in mitochondrial dynamics remained speculative. Mitochondrial nucleoids, enriched for TFAM and mtDNA, are frequently located near mitochondrial fission sites that coincide with mitochondria–ER contact sites (MERCS) [36]. These regions are thought to couple mtDNA replication with organelle division and recruit Drp1 for fission initiation [7, 36]. However, direct evidence linking TFAM to the core fission machinery was lacking. TFAM-promoted fragmentation occurred without altering mitochondrial number, ROS levels, membrane potential, mass, or mtDNA copy number (Figs. 1L and S2A–D), indicating morphological changes result specifically from enhanced fission rather than secondary effects. Fluorescence co-localization demonstrated that TFAM-mediated fission depends on Drp1 (Fig. 3H). Knockdown of MFF and Drp1, plus pharmacological inhibition of AMPK and Drp1 (Figs. 3A–G and S4A, B), confirmed that TFAM promotes fission through the AMPK/MFF/Drp1 pathway. These findings identify a mechanistic link between TFAM and mitochondrial fission regulation.

Our findings that TFAM increases the mitochondrial enrichment of Sirt3 and Sirt3-dependent mitochondrial protein deacetylation (Figs. 5A–D and S6A–F; S7C), and that pharmacological inhibition or genetic silencing of Sirt3 attenuates TFAM-induced phosphorylation of AMPK, MFF, and mitochondrial Drp1 (Figs. 5F, G and S6G–L), identify Sirt3 as a key molecular link connecting TFAM to the AMPK/MFF/Drp1 pathway. Evidence suggests that Sirt3-mediated AMPK activation occurs through deacetylation of liver kinase B1 (LKB1) [10, 12]. The primary upstream kinase that phosphorylates AMPK at Thr172 [37, 38]. We propose that TFAM-dependent mitochondrial enrichment of Sirt3 to mitochondria leads to LKB1 deacetylation and activation, which in turn promotes phosphorylation of AMPK at Thr172, triggering the downstream MFF/Drp1 mitochondrial fission pathway. Traditionally, Sirt3 has been positioned upstream of TFAM, regulating TFAM’s function by enhancing TFAM stability and mtDNA binding [39, 40], and in sepsis-associated kidney injury, Sirt3 deacetylates TFAM at K154 to enhance mitophagy [10]. Our data reveal a reciprocal relationship, with TFAM also acting upstream of Sirt3. TFAM overexpression increased mitochondrial Sirt3 abundance and Sirt3-dependent mitochondrial deacetylation (Fig. 5A–E), while Sirt3 inhibition impaired TFAM-promoted fission (Fig. 5F–K). Co-immunoprecipitation and live-cell FRET confirmed direct TFAM-Sirt3 interaction (Fig. 6B, C). Immunofluorescence and mitochondrial-fraction Western blotting showed marked enrichment of Sirt3 in mitochondria, with only a modest increase in total Sirt3 levels (Figs. 5A, B and S6A). Quantitative PCR, however, showed no change in SIRT3 mRNA (Fig. S6B). Together with previous reports that Sirt3 carries an N-terminal presequence and is imported into the mitochondrial matrix through the TOM-TIM23 pathway in a membrane-potential–dependent manner [41, 42]. These findings are consistent with a “stabilization and retention” model in which Sirt3 is imported via the canonical machinery and then stabilized by interaction with TFAM in the matrix.

Our observations that targeted mutagenesis of TFAM’s HMG-box A domain abolishes the interaction of TFAM with Sirt3 and eliminates the downstream effects of TFAM on mitochondrial dynamics (Figs. 6F–J and S8) indicate that HMG-box A serves as the key structural interface mediating the regulatory role of TFAM in mitochondrial fission via Sirt3. The HMG-box A domain traditionally mediates mtDNA binding and nucleoid packaging [43–45]. TFAM-Mut1 exhibited reduced FRET with Sirt3 (Fig. 6I), failed to recruit Sirt3 to mitochondria (Fig. 6J), increased mitochondrial protein acetylation (Fig. 6H), and showed reduced capacity to promote fragmentation (Fig. 6F, G). We propose that HMG-box A functions as a molecular switch dependent on DNA-binding state: when DNA-bound, the domain is occluded from protein interactions; when DNA-free, HMG-box A serves as a platform for Sirt3 binding. DNA binding physically occludes lysine residues, reducing accessibility to modifying enzymes [35]. Lu et al. further showed that phosphorylation of TFAM within the HMG-box A impairs the ability to bind DNA and activate transcription, and only DNA-free TFAM is susceptible to degradation by the Lon protease [34]. Taken together, these findings support a model in which the HMG-box A functional state—DNA-bound versus free—dictates TFAM function, stability, and turnover, coordinating mtDNA maintenance with mitochondrial dynamics. Further studies are needed to elucidate structural determinants and the role of post-translational modifications in this switch mechanism.

Our analyses show that high TFAM-SIRT3 mRNA co-expression is associated with overall survival, particularly in KIRC (Fig. 7D), and provide clinical association evidence consistent with possible disease relevance of the TFAM-Sirt3-AMPK/MFF/Drp1 pathway. Although TFAM and SIRT3 transcripts are positively correlated across tumors, only TFAM is downregulated in KIRC relative to normal tissue (Fig. 7B, C), suggesting that TFAM acts as an upstream post-transcriptional regulator of SIRT3 by controlling its mitochondrial recruitment rather than SIRT3’s mRNA expression. Our mechanistic findings support a model wherein disruption of the TFAM-Sirt3-AMPK/MFF/Drp1 pathway may be linked to tumor biology in tumors such as KIRC through two potential mechanisms. First, mitochondrial fission facilitates apoptosis, partly by ensuring the efficient release of cytochrome [46, 47]. TFAM reduction in KIRC may impair pro-apoptotic fission, potentially enabling cancer cells to evade programmed cell death [48, 49]. Second, excessive mitochondrial elongation has been linked to glycolytic reprogramming and increased metastatic potential [50, 51]. A functional TFAM-SIRT3 axis that maintains fission capacity might counteract metabolic shifts associated with tumor growth and invasion [52, 53]. We therefore propose that the prognostic significance of the TFAM-SIRT3 signature reflects the functional status of mitochondrial dynamics, and that selective TFAM downregulation in KIRC (Fig. 7C) may weaken the TFAM-SIRT3 protective axis and be associated with poor outcome.

Collectively, our work identifies a TFAM-Sirt3-AMPK/MFF/Drp1 mechanism that promotes mitochondrial fission. Zebrafish TEM supports the ultrastructural phenotype in vivo (Fig. 2), but genetic validation in rodent and other mammalian models remains an important limitation of the present study. Future work in these models and patient samples will be necessary to establish tissue-level consequences and disease relevance.

## Supplementary information

Supplementary_Figures_and_Tables

Uncropped_Blots

## Data Availability

All data generated or analyzed during this study are included in this paper and will be made available on request.
