# Supplementary material for: TFAM promotes mitochondrial division by increasing mitochondrial Sirt3
Source: Cell Death Dis. 2026 Apr 21;17(1):525. doi: 10.1038/s41419-026-08750-w (PMC13230541; doi:10.1038/s41419-026-08750-w)
Supplement: Supplementary file 1 — Supplementary_Figures_and_Tables [file 41419_2026_8750_MOESM1_ESM.docx]

**Supplementary material for**

**TFAM promotes mitochondrial division by increasing mitochondrial Sirt3**

Table of contents

Fig. S1....................................................................................................2

Fig. S2....................................................................................................3

Fig. S3....................................................................................................4

Fig. S4....................................................................................................5

Fig. S5....................................................................................................6

Fig. S6....................................................................................................7

Fig. S7....................................................................................................8

Fig. S8....................................................................................................9

Table. S1.................................................................................................10

Table. S2.................................................................................................11

Table. S3.................................................................................................12

Table. S4.................................................................................................14


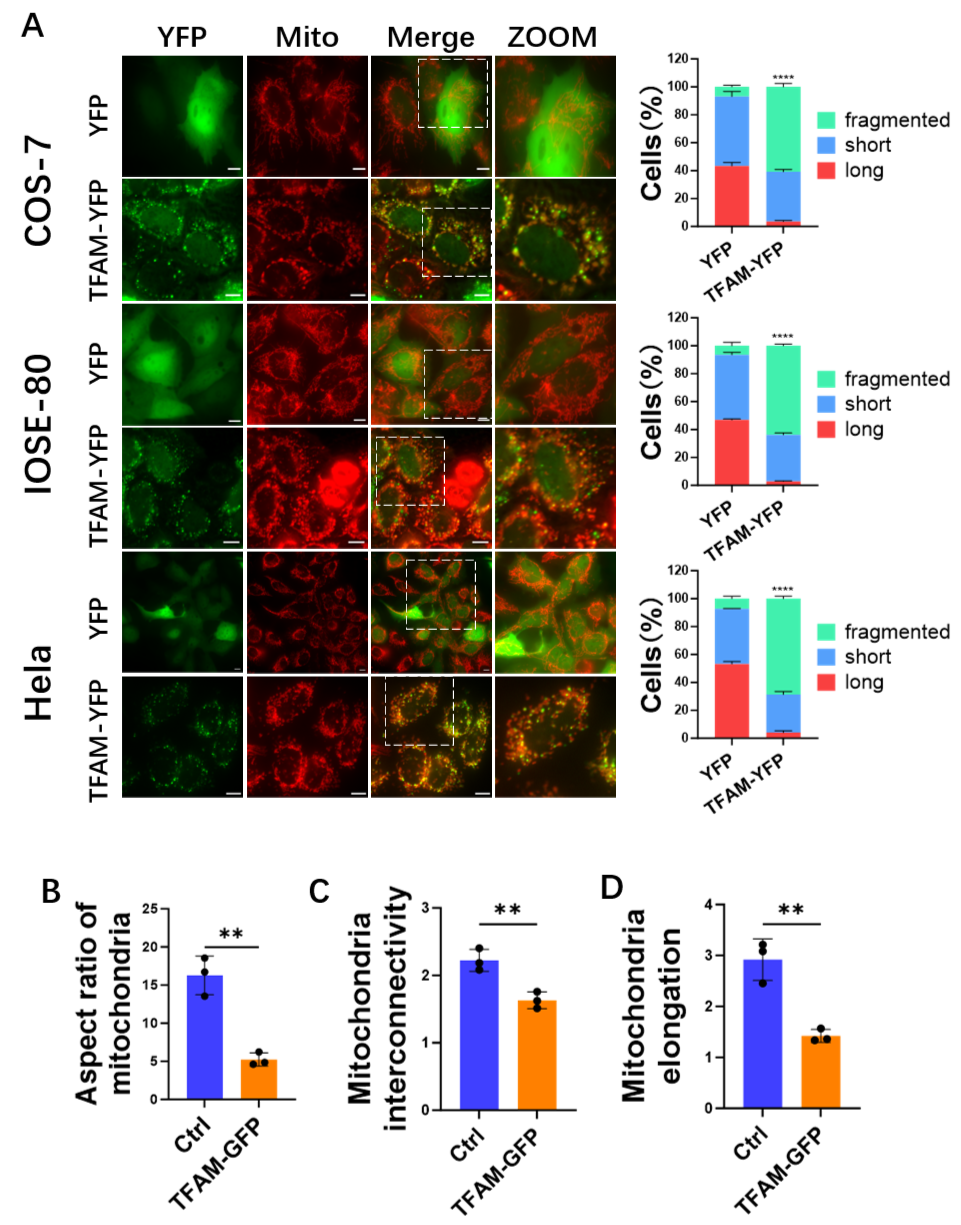


**Fig. S1 TFAM overexpression induces mitochondrial fragmentation across multiple cell lines and quantitative morphological analysis.** (A) Representative fluorescence images of cells expressing YFP or TFAM–YFP (green) and mitochondria labeled with MitoTracker (red), with merged and enlarged views. Bar graphs quantify the percentage of cells with fragmented, short, or long mitochondria; in each independent experiment, n = 100 cells were analyzed per condition. Statistical significance was assessed by Student’s t-test; ****P < 0.0001. (B–D) Quantitative analysis of mitochondrial morphological parameters corresponding to Fig. 1G. (B) Mitochondrial aspect ratio. (C) Mitochondrial interconnectivity. (D) Mitochondrial elongation. Bar graphs show mean ± SD of three independent experiments; n = 180 mitochondria from 60 cells were analyzed per condition. Statistical significance was determined by Student’s t-test; **P < 0.01.


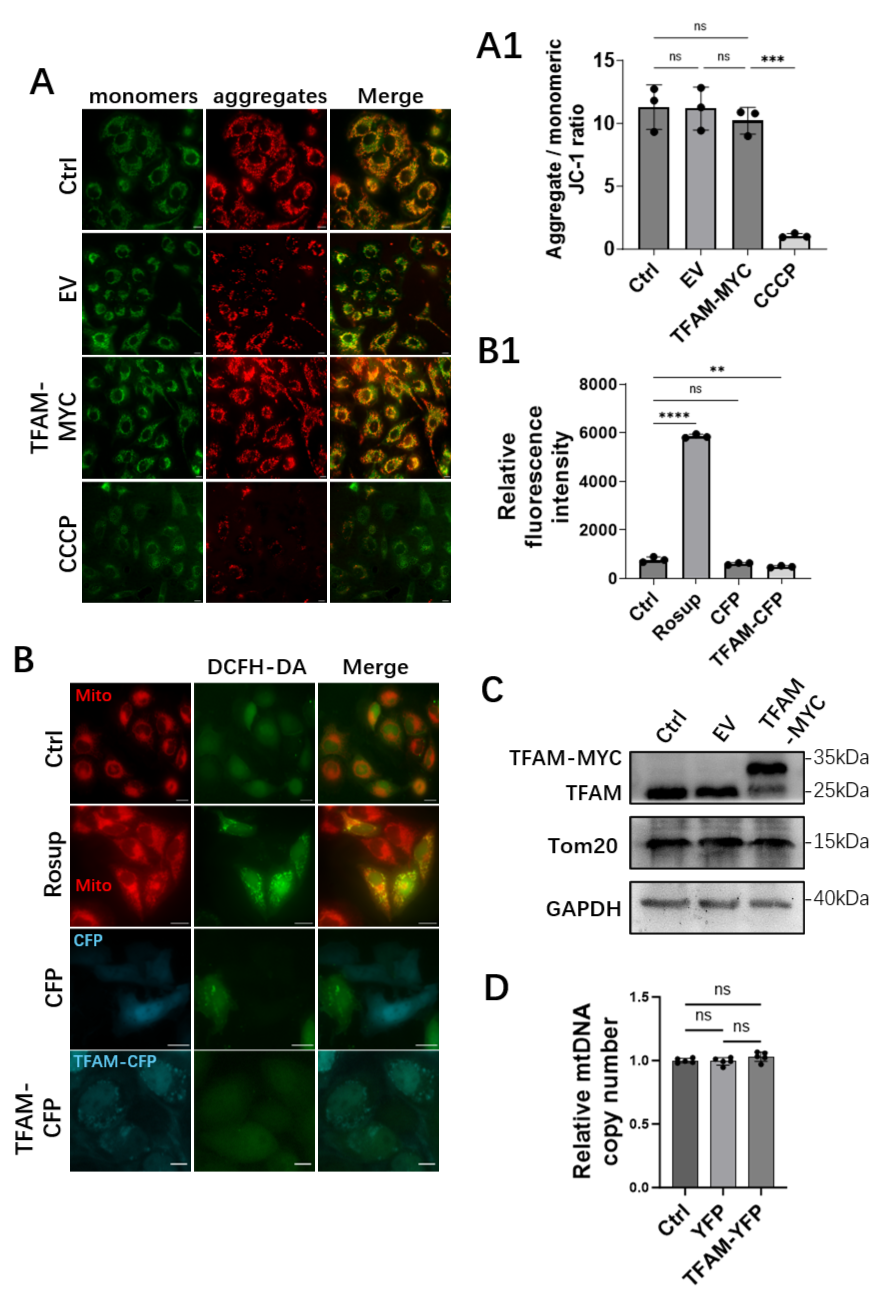


**Fig. S2 TFAM overexpression does not alter key indicators of mitochondrial health and biogenesis.** (A) Representative JC-1 staining of mitochondrial membrane potential (ΔΨm). Red aggregates indicate high potential and green monomers low potential; CCCP was used as a positive control. (A1) Quantification of the aggregate/monomer JC-1 fluorescence ratio (mean ± SD, n = 3; 50 cells per condition in each experiment). (B) Detection of intracellular ROS with DCFH-DA (green) in cells expressing CFP or TFAM–CFP; Rosup was used as a positive control. (B1) Quantification of relative DCFH-DA fluorescence intensity (mean ± SD, n = 3; 50 cells per condition in each experiment). (C) Representative Western blot of TFAM–MYC, endogenous TFAM, and the mitochondrial mass marker Tom20 (n = 3). (D) Relative mtDNA copy number measured by qPCR in cells expressing YFP or TFAM–YFP (mean ± SD, n = 5). Scale bars, 10 μm. Statistical analysis in (A1, B1, D) was performed using one-way ANOVA. ****P < 0.0001; ns, not significant. n, number of independent experiments.


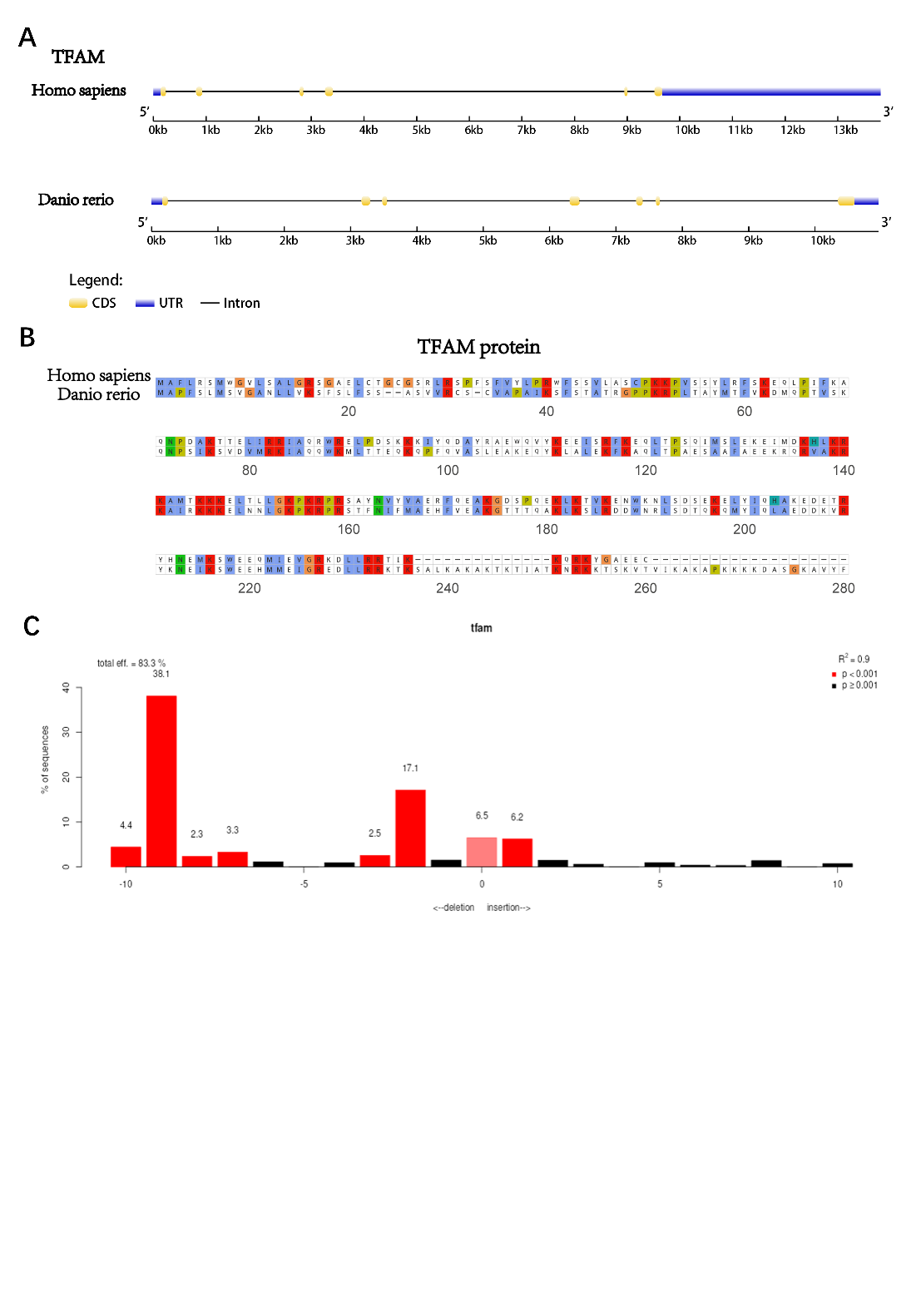


**Fig. S3 TFAM conservation and disruption in zebrafish.** (A) Genomic organization of the TFAM locus in Homo sapiens and Danio rerio, showing exon–intron structure and untranslated regions (UTRs) on a kilobase scale. The human locus spans ~13 kb with 6 exons, whereas the zebrafish locus spans ~10 kb with 7 exons. (B) Protein sequence alignment of human and zebrafish *TFAM*. Amino acids are colored by biochemical properties, identical residues are boxed in red, and HMG-box domains are indicated. Overall similarity is 68% (BLAST E-value = 7 × 10⁻⁵⁴; query coverage 83%). (C) Indel spectrum at the tfam locus in 24 hpf embryos (pooled n = 20), indicating efficient *tfam* disruption.


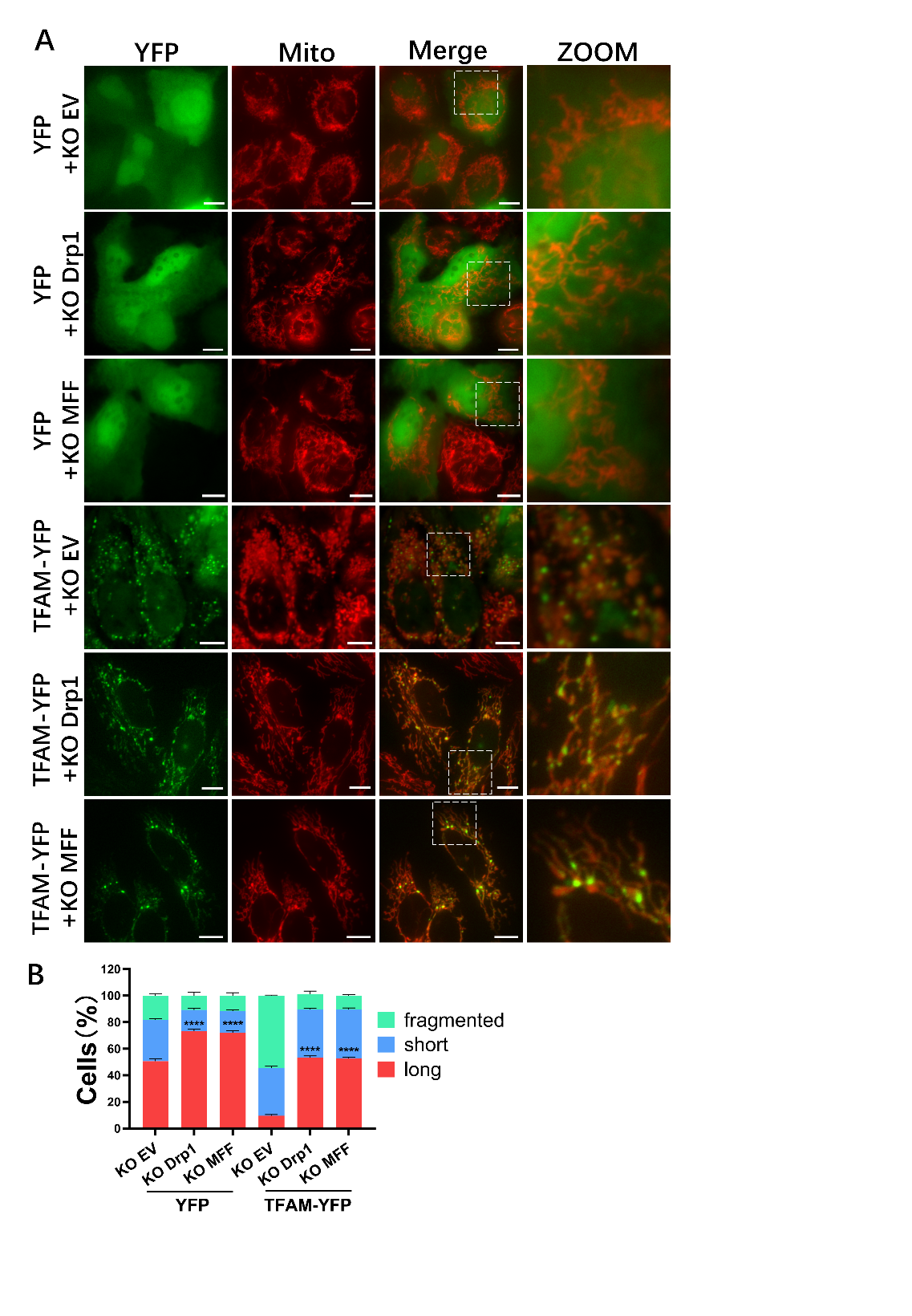


**Fig. S4 Mitochondrial morphology in control, Drp1-KO, and MFF-KO cells expressing YFP or TFAM-YFP.** (A) Representative live-cell fluorescence images of mitochondria in MCF-7 cells expressing YFP (EV) or TFAM–YFP under different knockout backgrounds: control (EV), Drp1 knockout (Drp1-KO), or MFF knockout (MFF-KO). Images show YFP (green), mitochondria (MitoTracker, red), merged channels, and zoomed views (dashed boxes). Scale bars, 10 μm. (B) Quantification of mitochondrial morphology from (A). Data are presented as mean ± SD (n = 3); n = 100 cells were analyzed per condition. Statistical significance was determined by one-way ANOVA. Significance is indicated as ****P < 0.0001.


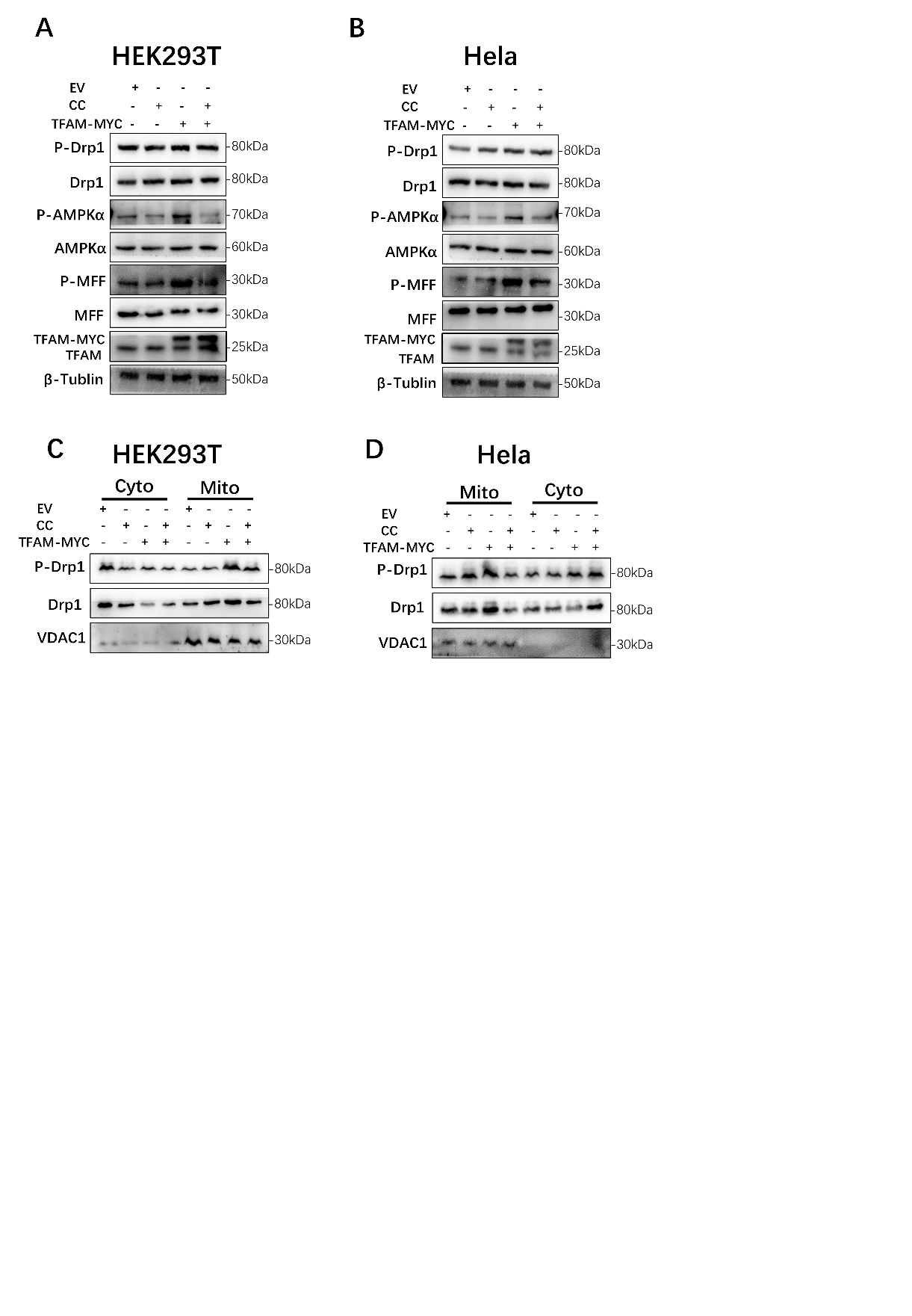


**Fig. S5 AMPK/MFF/Drp1 pathway analysis in HEK293T and HeLa cells.** (A, B) Western blot analysis of whole-cell lysates from HEK293T (A) and HeLa (B) cells. (C, D) Western blot analysis of cytoplasmic and mitochondrial fractions from HEK293T (C) and HeLa (D) cells. Cells were transfected with EV or TFAM–MYC and treated with or without CC (20 μM, 6 h). All blots are representative of three independent experiments.


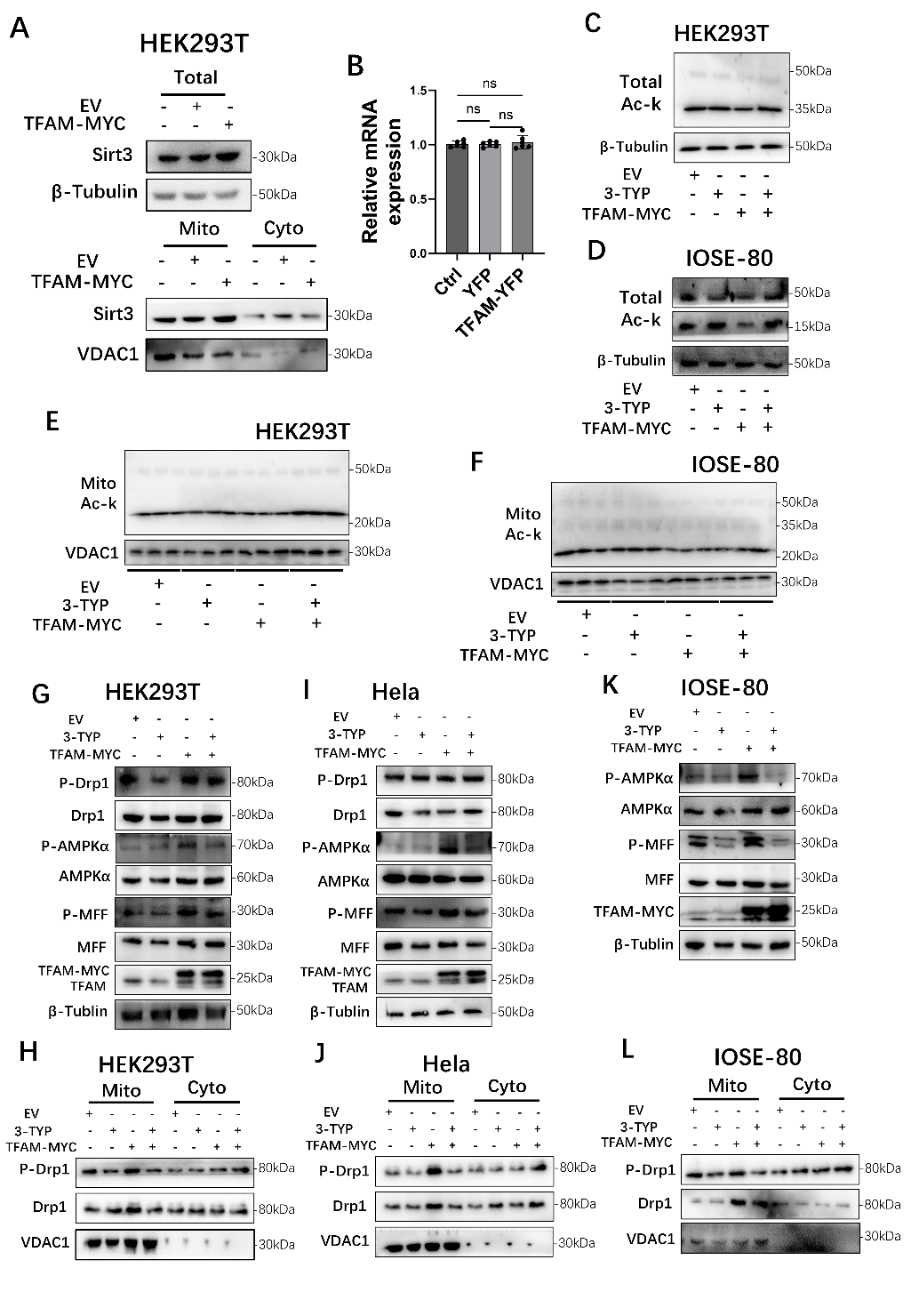


**Fig. S6 Validation of TFAM effects on Sirt3 and downstream signaling in multiple cell lines.** (A) Western blot analysis of Sirt3 levels in total cell lysates and in mitochondrial and cytoplasmic fractions. (B) qPCR analysis of SIRT3 mRNA in three conditions: Ctrl, YFP (EV overexpression), and TFAM–YFP (TFAM overexpression) (n = 5). Expression was normalized to GAPDH and plotted relative to Ctrl (= 1). Bars show mean ± SD; ns, not significant by one-way ANOVA. (C–F) Protein acetylation levels in cells transfected with EV or TFAM–MYC and treated with or without 3-TYP (10 μM, 12 h). (G–L) Phosphorylation levels of AMPK, MFF, and Drp1 in mitochondrial fractions from cells transfected with EV or TFAM–MYC and treated with or without 3-TYP (10 μM, 12 h). All blots are representative of three independent experiments.


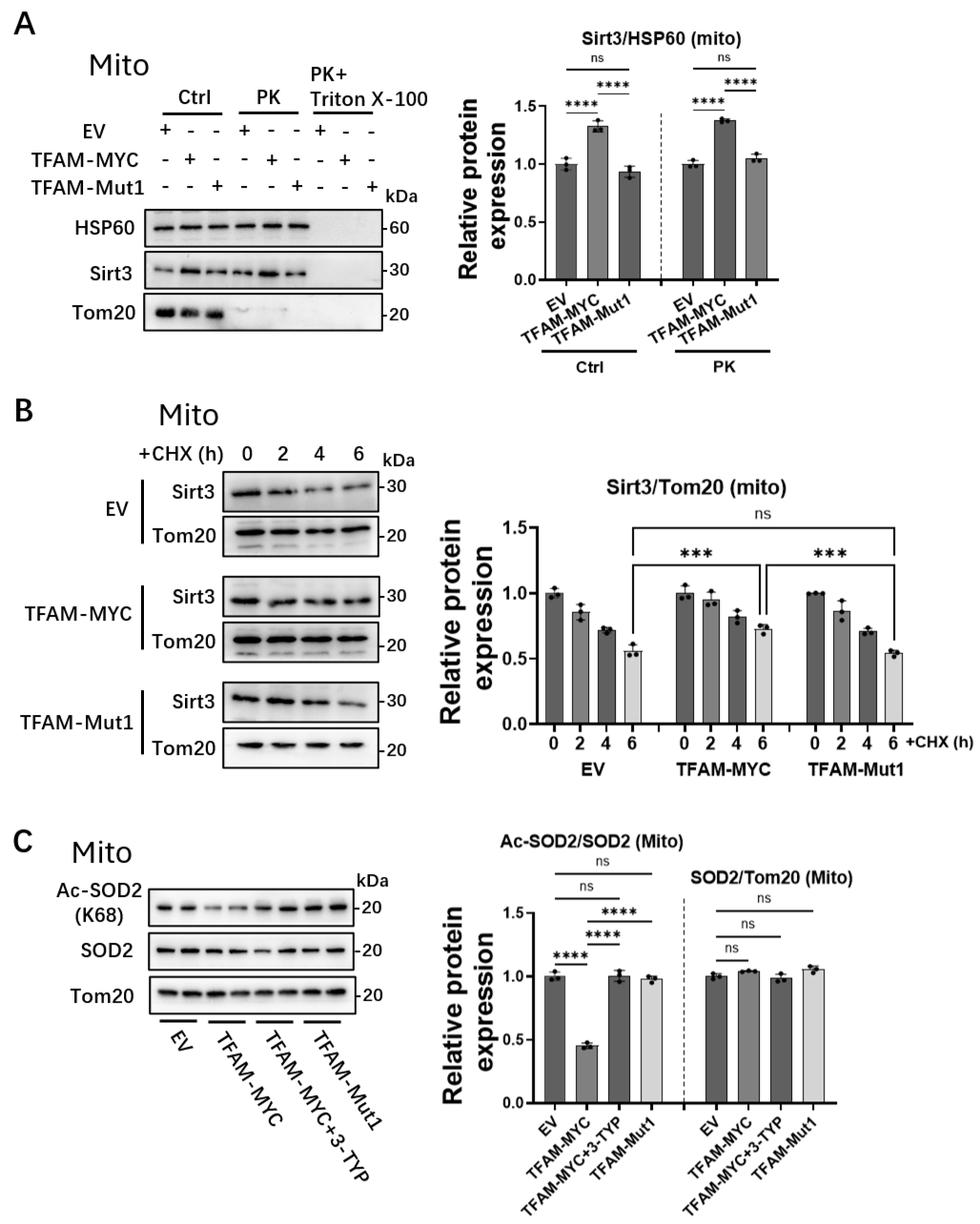


**Fig. S7 TFAM promotes intramitochondrial enrichment and functional deacetylation readouts of Sirt3.** (A) Proteinase K (PK, 50 μg/mL) protection assay in isolated mitochondria from HEK293T cells transfected with empty vector (EV), TFAM-WT, or TFAM-Mut1. Mitochondria were left untreated (No PK), treated with PK in the absence of detergent, or treated with PK in the presence of Triton X-100, followed by immunoblotting for Sirt3 and the indicated mitochondrial marker proteins. (B) Cycloheximide (CHX, 75 μg/mL) chase assay. HEK293T cells expressing EV, TFAM-WT, or TFAM-Mut1 were treated with CHX and mitochondria were collected at the indicated time points for immunoblot analysis of mitochondrial Sirt3. (C) Immunoblot analysis of acetylated SOD2 at lysine 68 (Ac-SOD2 K68) and total SOD2 in mitochondrial fractions from HEK293T cells expressing EV, TFAM-WT, or TFAM-Mut1, with or without 3-TYP treatment, as indicated. Data are presented as mean ± SD from three independent experiments. One-way ANOVA was used for statistical analysis. ****P < 0.0001, ***P < 0.001, ns, not significant.


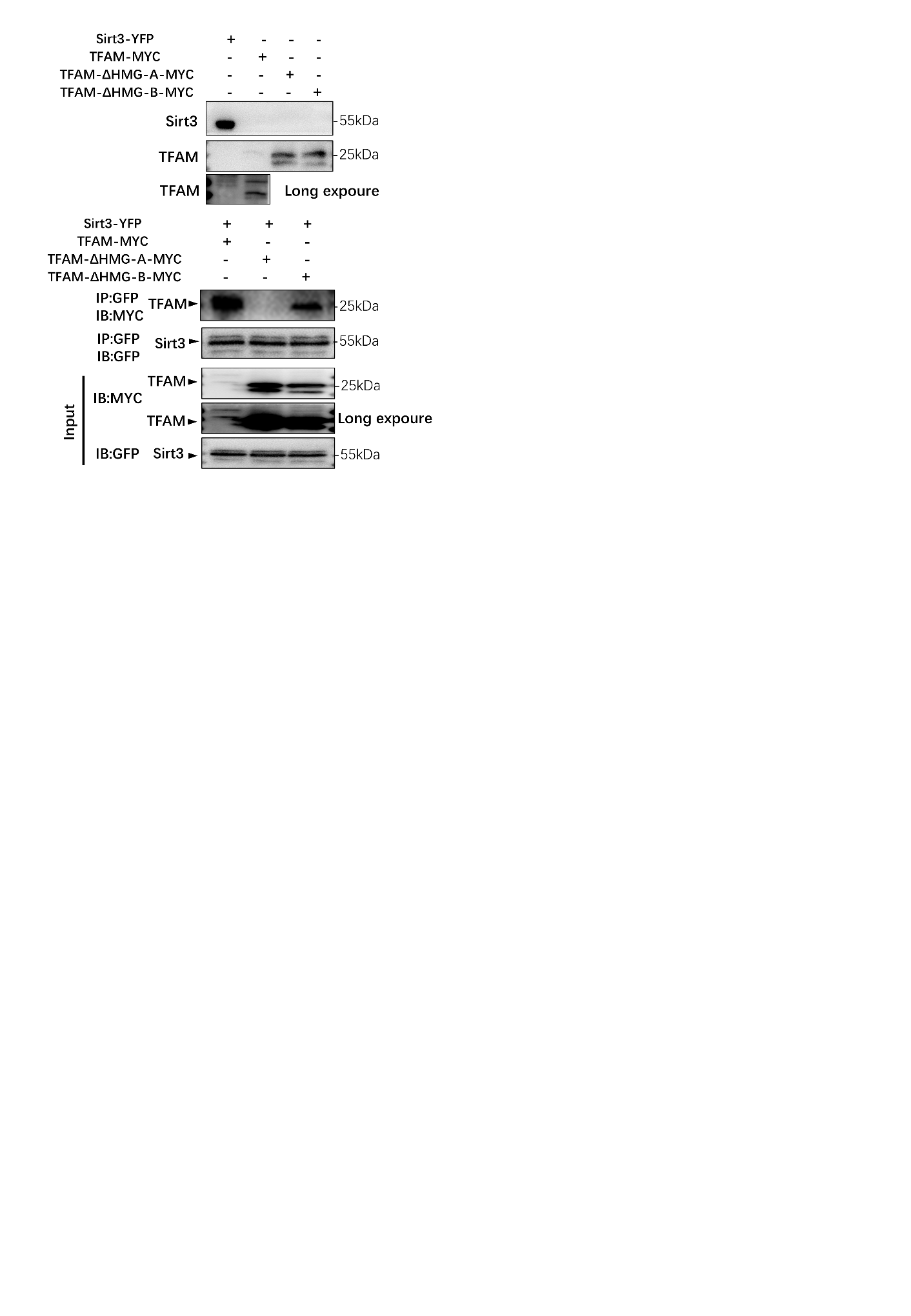


**Fig. S8 Co-immunoprecipitation of Sirt3–YFP with TFAM deletion mutants.** Cell lysates from cells expressing Sirt3–YFP together with TFAM variants (WT, ΔHMG-box A, ΔHMG-box B) were immunoprecipitated with anti-GFP antibody and immunoblotted with anti-MYC antibody to detect TFAM. All blots are representative of three independent experiments.

**Table S1: Antibodies**

| **Name** | **Supplier** | **Cat. no.** |
| --- | --- | --- |
| Phospho-DRP1 (Ser616) (P-Drp1) | Cell Signaling Technology | 3455 |
| DRP1 | Cell Signaling Technology | 8579 |
| Tom20 | Cell Signaling Technology | 42406T |
| MFF | Affinity Biosciences | DF12006 |
| Phospho-MFF (P-MFF) | Affinity Biosciences | AF2365 |
| Drp1 | Cell Signaling Technology | 8570 |
| SIRT3 (SirT3) | Cell Signaling Technology | 2627 |
| GAPDH | Cell Signaling Technology | 2118 |
| AMPKα1 (AMPK) | Cell Signaling Technology | 2795T |
| Phospho-AMPKα1 (T172) (P-AMPK) | Cell Signaling Technology | 8208 |
| β-Tubulin | Cell Signaling Technology | 2146 |
| GFP | Cell Signaling Technology | 2956 |
| MYC-tag | Cell Signaling Technology | 2276 |
| TFAM | Santa Cruz Biotechnology | sc-166965 |
| VDAC1 | Santa Cruz Biotechnology | sc-390996 |
| Acetyl-lysine (Ac-K) | Santa Cruz Biotechnology | sc-32268 |
| SOD2 (acetyl K68) | Abcam | ab137037 |
| SOD2 | Abcam | ab68155 |
| HRP-conjugated secondary antibodies | Beyotime Biotechnology | A0210/ A0218 |
| Alexa Fluor–conjugated secondary antibodies | Beyotime Biotechnology | A0423/ A0453 |

**Sequence-based reagents**

| **Name** | **Sequence (5'–3')** |
| --- | --- |
| *tfam* sgRNA | TATGCTGTCAGGGGTCTCTT |

| **Table S2. Reagents (media, drugs, kits, dyes, etc.)** | | |
| --- | --- | --- |
| **Name** | **Supplier** | **Cat. no.** |
| Fetal bovine serum (FBS) | Thermo Fisher Scientific | A5256701 |
| DMEM (Dulbecco’s Modified Eagle Medium) | Thermo Fisher Scientific | 12491015 |
| McCoy’s 5A medium | Procell | PM150710 |
| PBS (Phosphate-buffered saline) | Thermo Fisher Scientific | 10010 |
| Penicillin–streptomycin (1%) | Procell | PB180120 |
| DMSO (Dimethyl sulfoxide) | Sigma-Aldrich | 67-68-5 |
| MitoTracker Deep Red FM | Thermo Fisher Scientific | A66440 |
| ExFect Transfection Reagent | vazyme | T101 |
| Mdivi-1 | MedChemExpress | HY-15886 |
| 3-TYP | MedChemExpress | HY-108331 |
| AICAR  (5-Aminoimidazole-4-carboxamide ribonucleotide) | MedChemExpress | HY-13417 |
| Compound C (CC) | MedChemExpress | HY-13418A |
| BCA Protein Assay Reagent | Tiangen | PA115-01 |
| PAGE Gel Preparation Kit 10% | Beyotime | P0012AC |
| Mitochondrial and cytosolic fractionation kit | Beyotime | C3601 |
| Polybrene | Sigma-Aldrich | H9268 |
| PVDF membranes | Millipore | IPVH00010 |
| BSA (bovine serum albumin) | Sigma-Aldrich | A7906 |
| Paraformaldehyde (4% PFA) | Beyotime Biotechnology | P0096 |
| Triton X-100 | Beyotime Biotechnology | P0096 |
| DAPI | Beyotime Biotechnology | C1005 |
| Protein A/G beads | MedChemExpress | HY-K0202 |
| ECL detection reagent | Thermo Fisher Scientific | 32209 |
| Phenol red (0.1%) | Sigma-Aldrich | P0290 |
|  |  |  |
|  |  |  |
|  |  |  |

**Table S3. Plasmids and viral vectors**

| **Plasmid** | **Backbone** | **Supplier** | **Cat. no.** | **Notes** |
| --- | --- | --- | --- | --- |
| pcDNA3-CFP | pcDNA3 | Addgene | #13030 | CFP expression vector |
| pcDNA3-YFP | pcDNA3 | Addgene | #13033 | YFP expression vector |
| mCherry-ActA | – | Gift from David W. Andrews [1] | – | Mitochondria-targeted mCherry |
| GFP-MFF | – | Addgene | #49153 | GFP-tagged MFF |
| pCMV-SIRT3(human)-ECFP-Neo (Sirt3-CFP) | pCMV-ECFP-Neo | MiaoLing Plasmid Platform | P62342 | SIRT3-CFP fusion |
| pCMV-EYFP-PRKAA1(human)-Neo (YFP-AMPK) | pCMV-EYFP-Neo | MiaoLing Plasmid Platform | P39017 | YFP-AMPK fusion |
| pLV3-U6-CopGFP-Puro (shCtrl) | Lentiviral shRNA vector | MiaoLing Plasmid Platform | – | Empty control vector |
| pU6-SIRT3(human)-shRNA1-EGFP-Puro (shSirt3) | Lentiviral shRNA vector | MiaoLing Plasmid Platform | P39793 | shRNA targeting SIRT3 |
| pLV3-U6-TFAM(human)-shRNA1-CopGFP-Puro (shTFAM#1) | Lentiviral shRNA vector | MiaoLing Plasmid Platform | P75514 | TFAM shRNA #1 |
| pLV3-U6-TFAM(human)-shRNA2-CopGFP-Puro (shTFAM#2) | Lentiviral shRNA vector | MiaoLing Plasmid Platform | P75510 | TFAM shRNA #2 |
| pLV3-U6-TFAM(human)-shRNA3-CopGFP-Puro (shTFAM#3) | Lentiviral shRNA vector | MiaoLing Plasmid Platform | P75509 | TFAM shRNA #3 |
| TFAM-CFP | pcDNA3-CFP | This study | – | TFAM fused to N-terminal CFP |
| TFAM-YFP | pcDNA3-YFP | This study | – | TFAM fused to N-terminal YFP |
| pLVX-TFAM-myc (TFAM-myc) | pLVX-Puro | This study (backbone from MiaoLing) | – | C-terminal myc-tagged TFAM |
| MFF-CFP | pcDNA3-CFP | This study | – | MFF-CFP fusion |
| SIRT3-YFP | pcDNA3-YFP | This study | – | SIRT3-YFP fusion |
| epiCRISPR vector | epiCRISPR | Gift from Dr. Wang [2] | – | CRISPR KO backbone |
| epiCRISPR-Drp1-KO | epiCRISPR | This study | – | Drp1 gRNA inserted |
| epiCRISPR-MFF-KO | epiCRISPR | This study | – | MFF gRNA inserted |
| TFAM-Mut1 (TFAM-Mut1-YFP) | TFAM-YFP | This study | – | W46F, R47I, K54E, Y61F, R62I in HMG-box A |
| TFAM-Mut2 (TFAM-Mut2-YFP) | TFAM-YFP | This study | – | R98E, K103E at A/B junction |
| TFAM-Mut3 (TFAM-Mut3-YFP) | TFAM-YFP | This study | – | R191E in C-terminal domain |
| MFF-S172D-CFP | MFF-CFP | This study | – | Phospho-mimetic mutant |
| MFF-S172A-CFP | MFF-CFP | This study | – | Non-phosphorylatable mutant |
| TFAM-ΔHMG-A | pCMV | MiaoLing Plasmid Platform | G88610 | TFAM(human)-del(46-118aa) |
| TFAM-ΔHMG-B | pCMV | MiaoLing Plasmid Platform | G88611 | TFAM(human)-del(155-219Aaa) |
| psPAX2 | Packaging  plasmid | MiaoLing Plasmid Platform | P0261 | Lentiviral packaging |
| pMD2.G | Packaging  plasmid | MiaoLing Plasmid Platform | P0262 | Lentiviral envelope (VSV-G) |

[1] A. Aranovich, Q. Liu, T. Collins, F. Geng, S. Dixit, B. Leber, D.W. Andrews, Differences in the Mechanisms of Proapoptotic BH3 Proteins Binding to Bcl-XL and Bcl-2 Quantified in Live MCF-7 Cells, Mol Cell 45 (2012) 754–763. https://doi.org/10.1016/j.molcel.2012.01.030.

[2] Y. Xie, D. Wang, F. Lan, G. Wei, T. Ni, R. Chai, D. Liu, S. Hu, M. Li, D. Li, H. Wang, Y. Wang, An episomal vector-based CRISPR/Cas9 system for highly efficient gene knockout in human pluripotent stem cells, Sci Rep 7 (2017) 2320. https://doi.org/10.1038/s41598-017-02456-y.

**Table S4. Software**

| **Software name** | **Provider** | **Version Details** |
| --- | --- | --- |
| ZEN | Carl Zeiss Microscopy GmbH | 3.4 (Blue etition) |
| ImageJ | NIH | 1.54f |
| Origin | OriginLab | 2025 |
| GraphPad Prism | GraphPad Software | 10.0 |
| PyMOL | Schrödinger, LLC | 3.0.3 |
| HDOCK server | HDOCK (HUST) | http://hdock.phys.hust.edu.cn/ |
| GEPIA2 | GEPIA2 web server | http://gepia2.cancer-pku.cn/ |
| GEPIA3 | GEPIA3 web server | https://gepia3.bioinfoliu.com/ |
