# Supplementary material for: TFAM promotes mitochondrial division by increasing mitochondrial Sirt3
Source: Cell Death Dis. 2026 Apr 21;17(1):525. doi: 10.1038/s41419-026-08750-w (PMC13230541; doi:10.1038/s41419-026-08750-w)

Corresponds to Figure 1A, TFAM

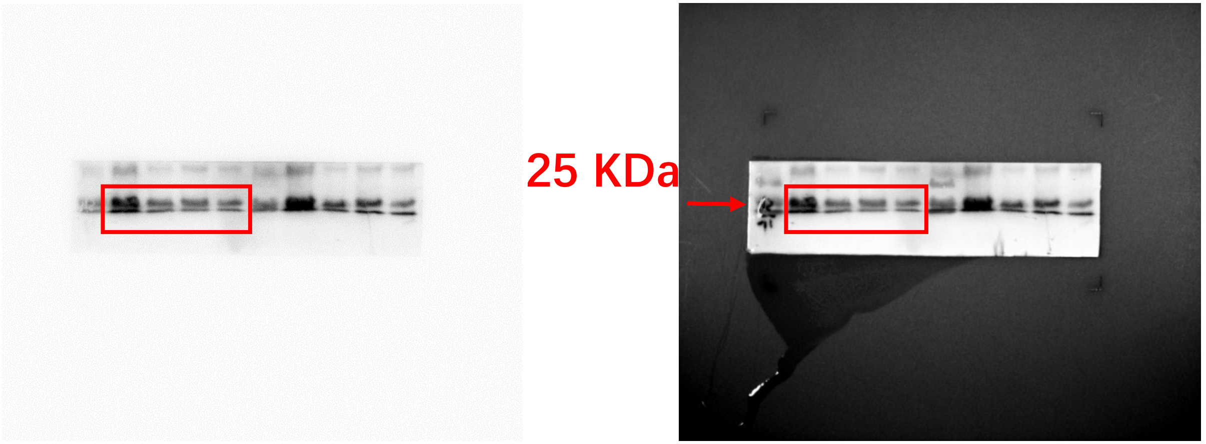

Corresponds to Figure 1A, Anti-β-Tubulin

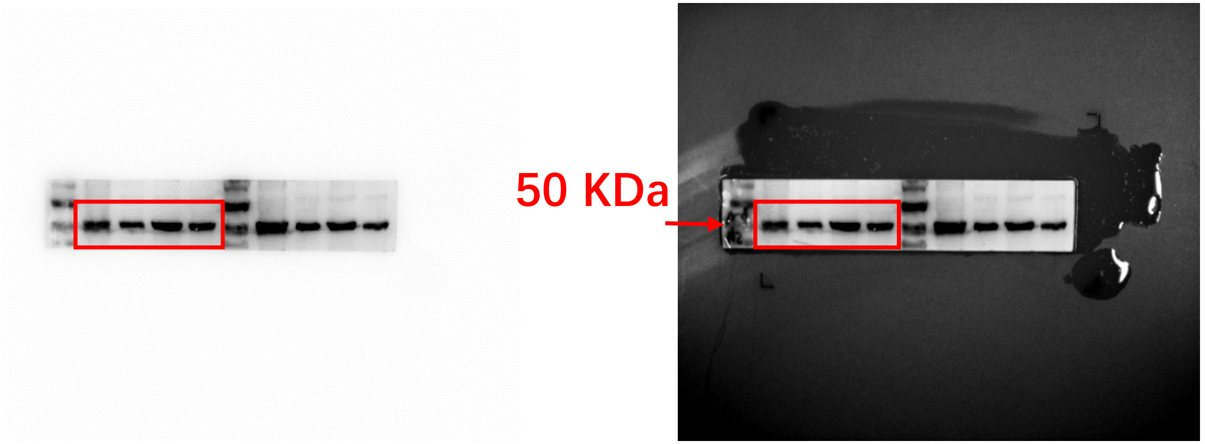

Corresponds to Figure 1D, Anti-MFF

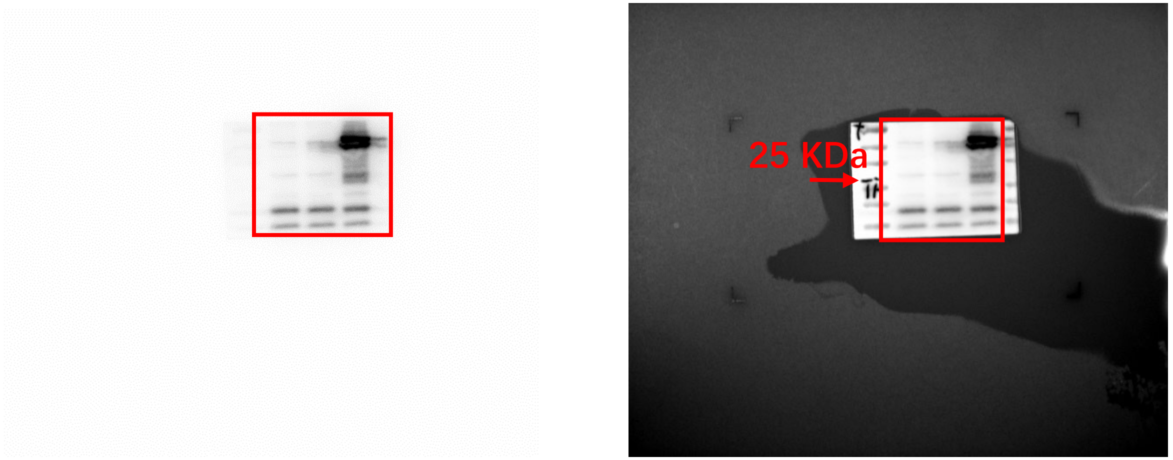

Corresponds to Figure 1D, Anti-GAPDH

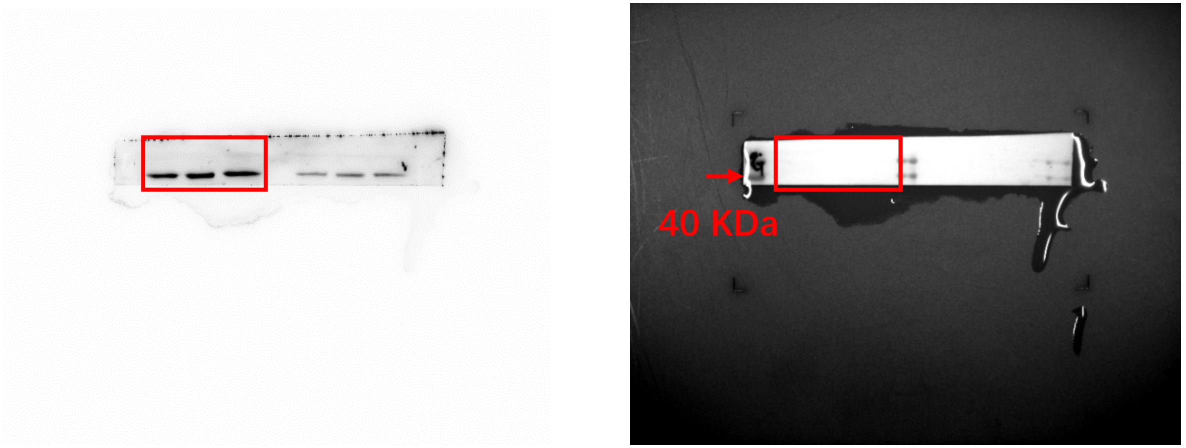

Corresponds to Figure 3A, Anti-β-Tubulin

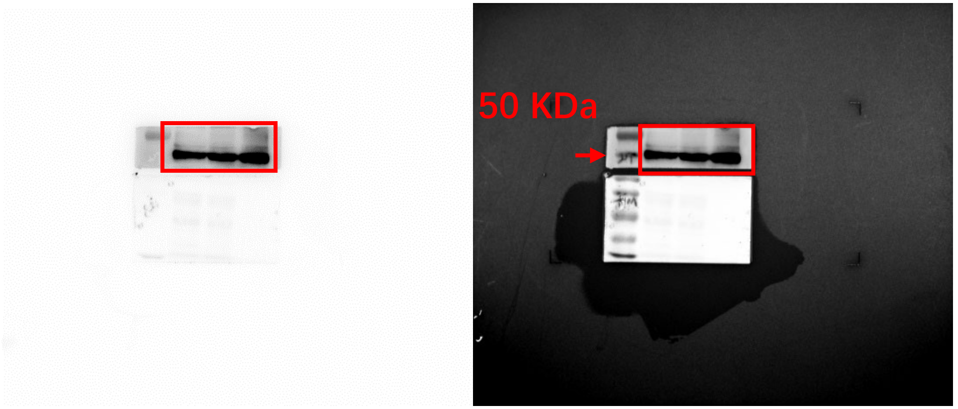

Corresponds to Figure 3A, Anti-MFF

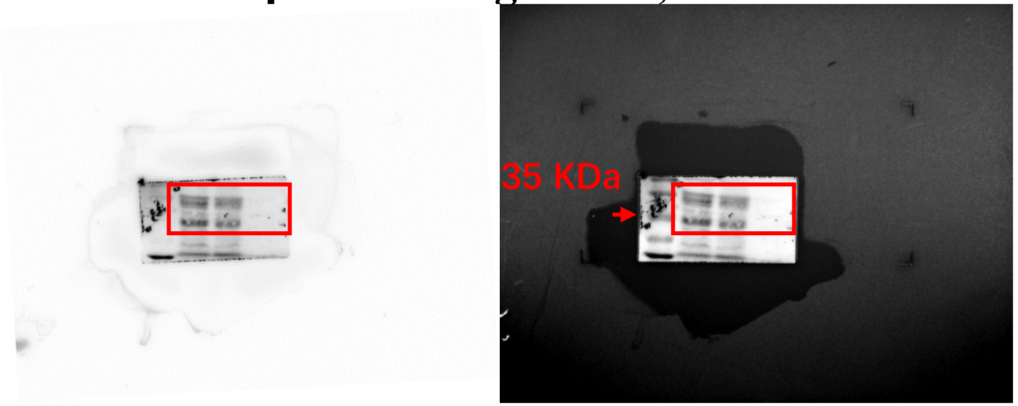

Corresponds to Figure 3A, Anti-GAPDH

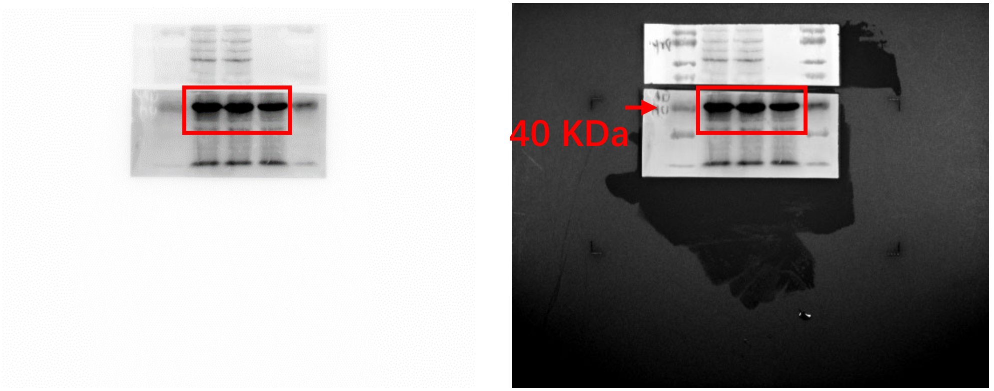

Corresponds to Figure 3A, Anti-Drp1

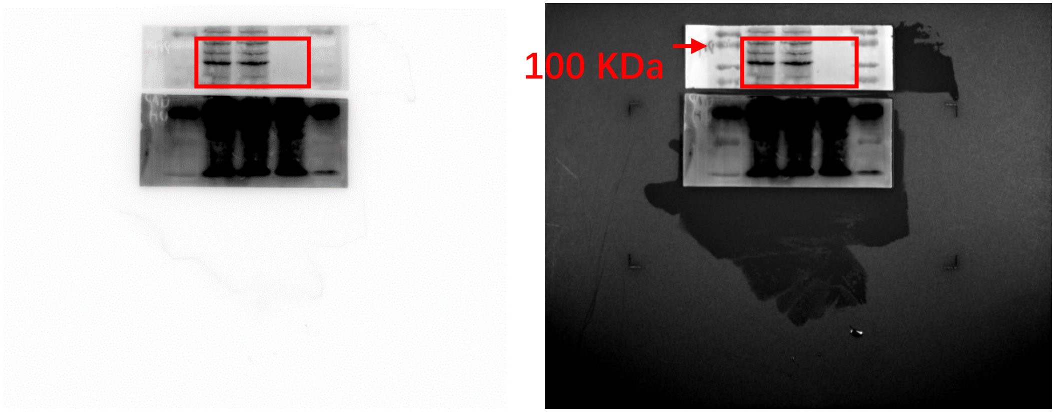

Corresponds to Fig. S2, Anti-Tomm20

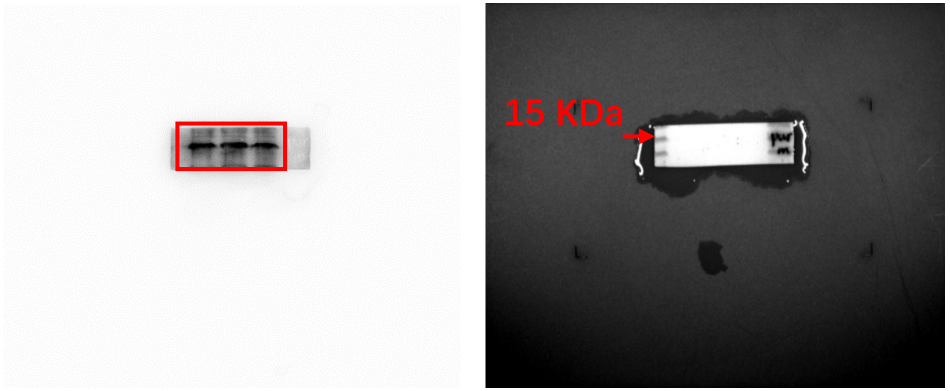

Corresponds to Figure 4A, Anti-GAPDH (left)
Corresponds to Fig. S2, Anti-GAPDH (right)

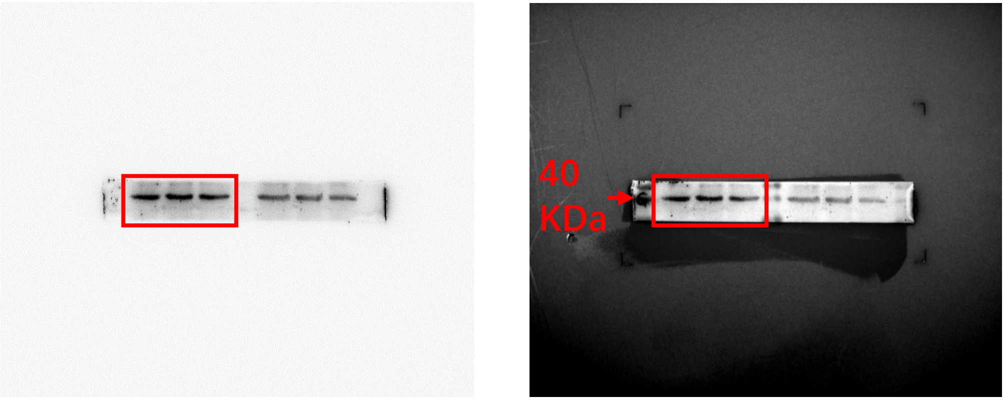

Corresponds to Figure 4A, Anti-TFAM (left)
Corresponds to Fig. S2, Anti-TFAM (right)

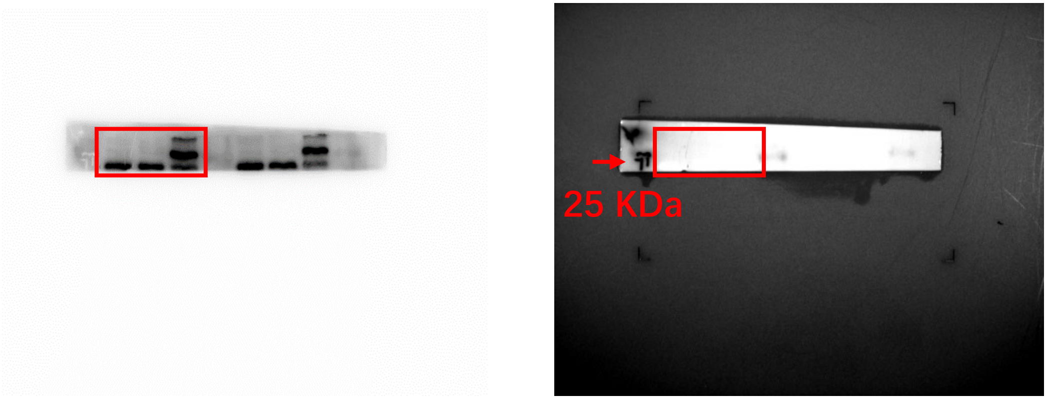

Corresponds to Figure 4B, Anti-AMPKα

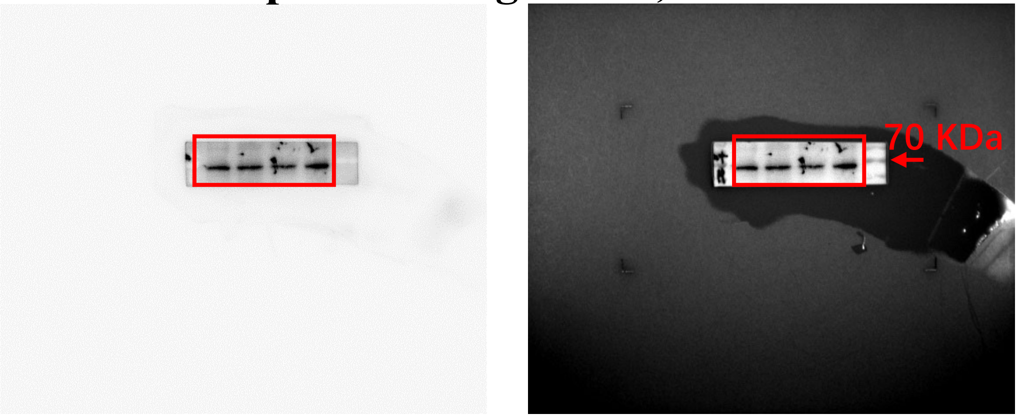

Corresponds to Figure 4B, Anti-P-AMPKα

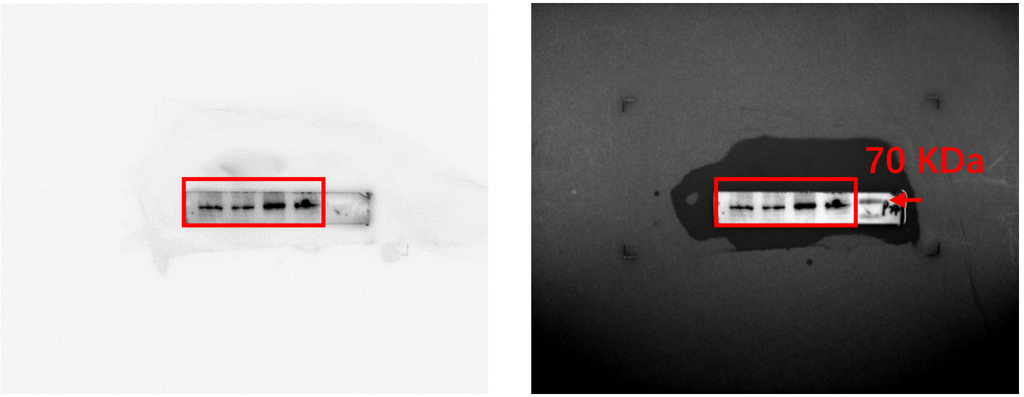

Corresponds to Figure 4B, Anti-P-MFF

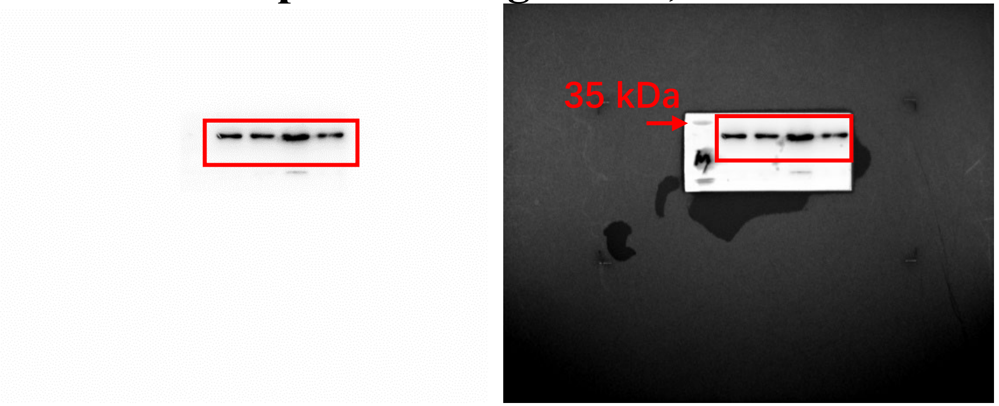

Corresponds to Figure 4B, Anti-MFF

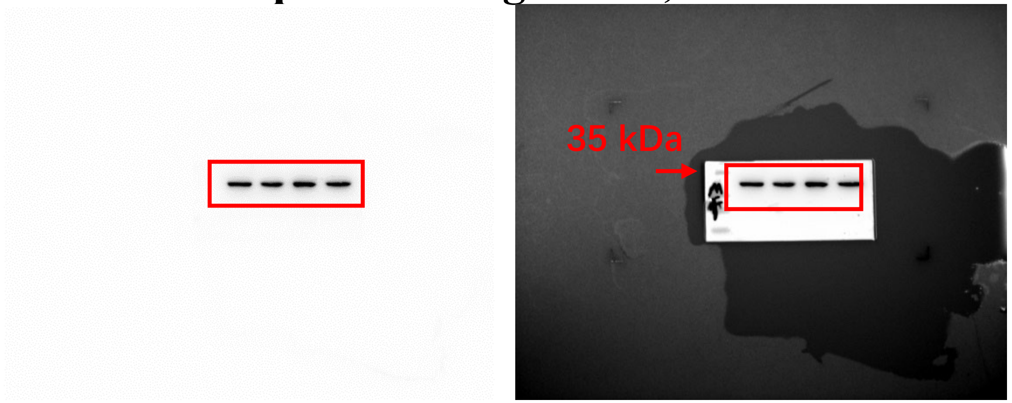

Corresponds to Figure 5B, bottom panel, Anti-β-Tubulin

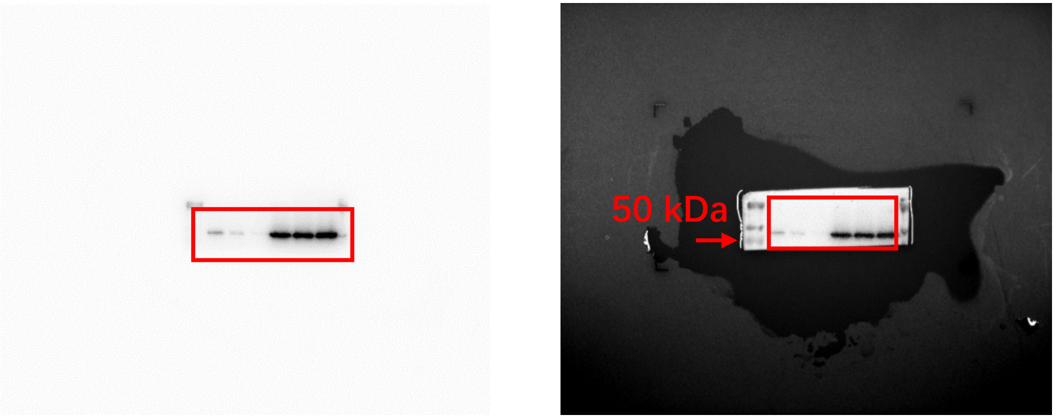

Corresponds to Figure 5B, Anti-GAPDH

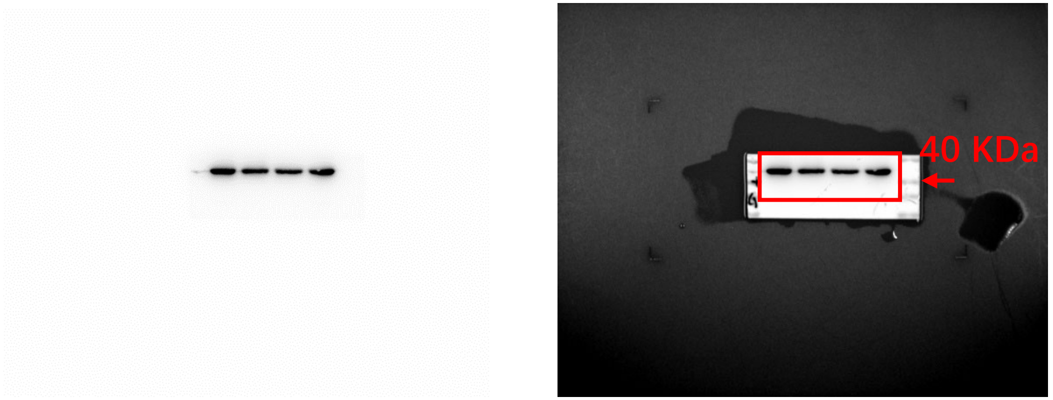

Corresponds to Figure 5B, upper panel, Anti-Sirt3

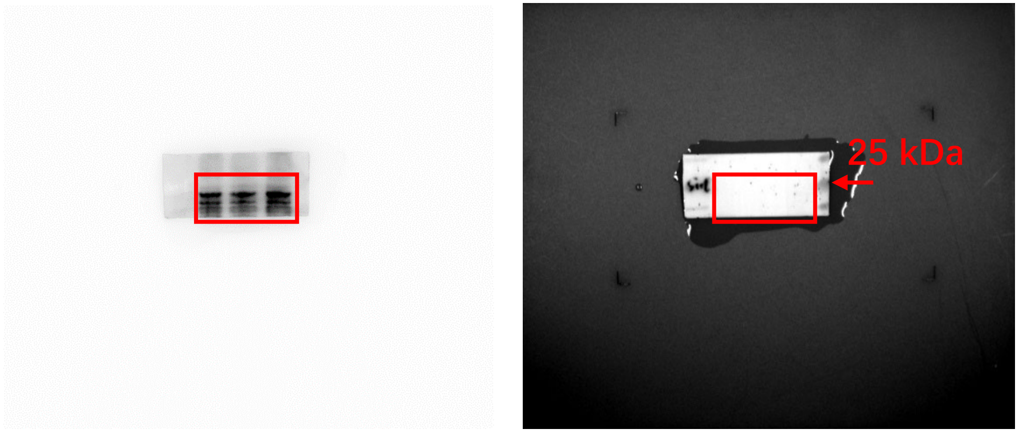

Corresponds to Figure 5B, upper panel, Anti-GAPDH

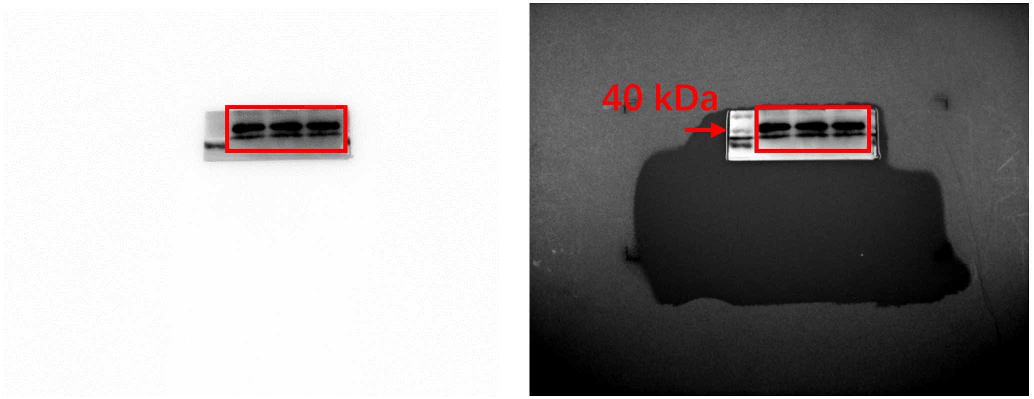

Corresponds to Figure 5B, lower panel, Anti-Sirt3

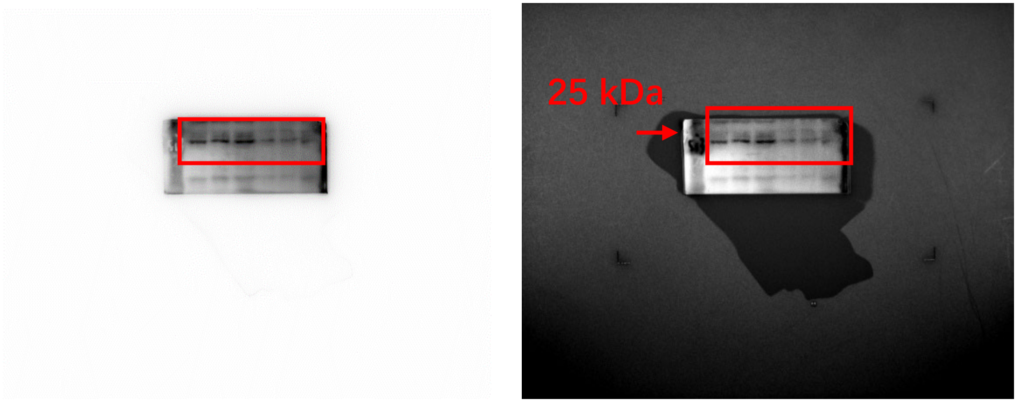

Corresponds to Figure 5B, lower panel, Anti-VDAC1

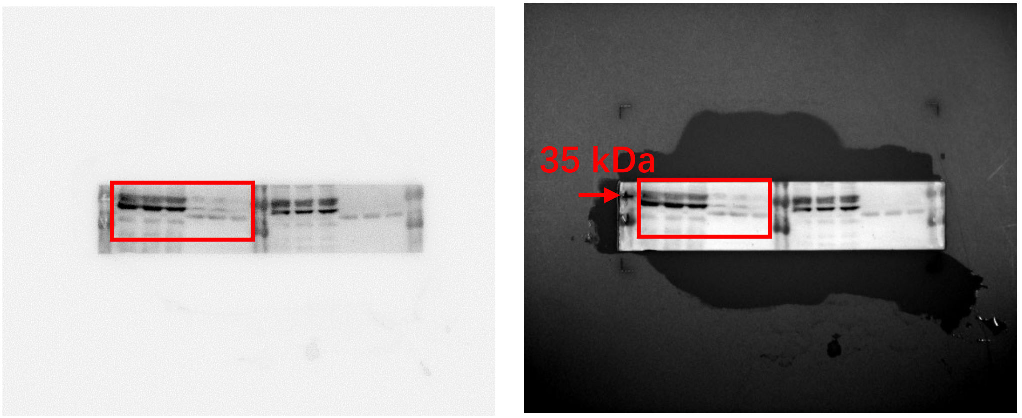

Corresponds to Figure 5C, Anti-Ac-k

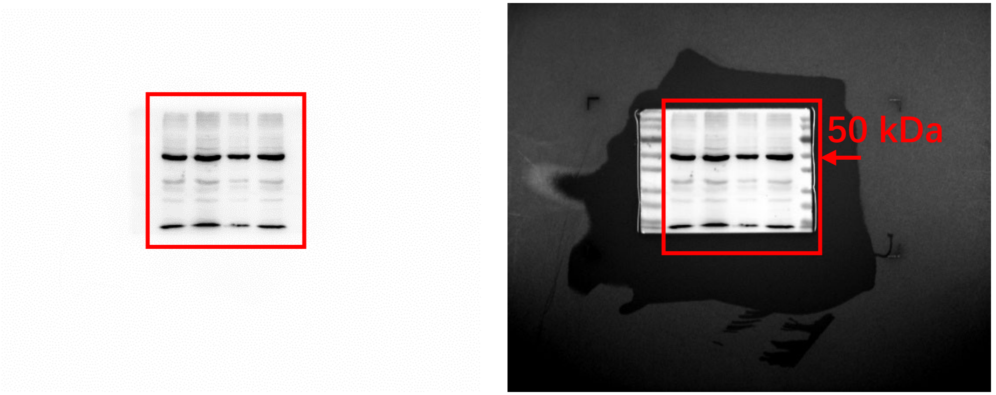

Corresponds to Figure 5D, Anti-Ac-k

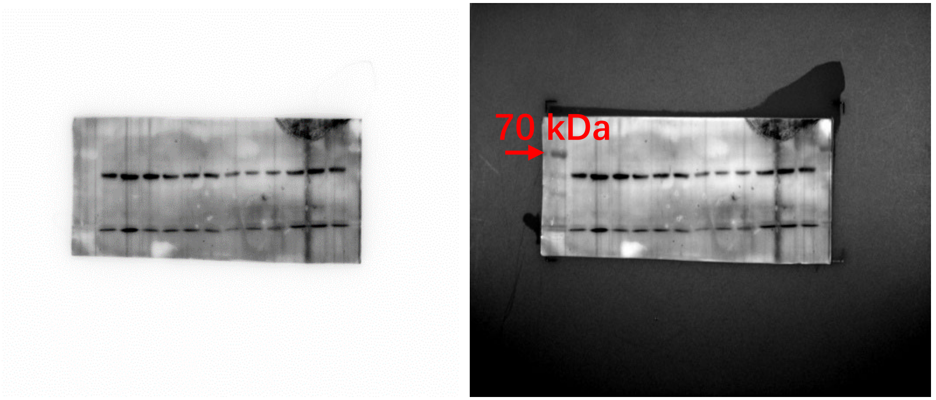

Corresponds to Figure 5E, Anti-Sirt3

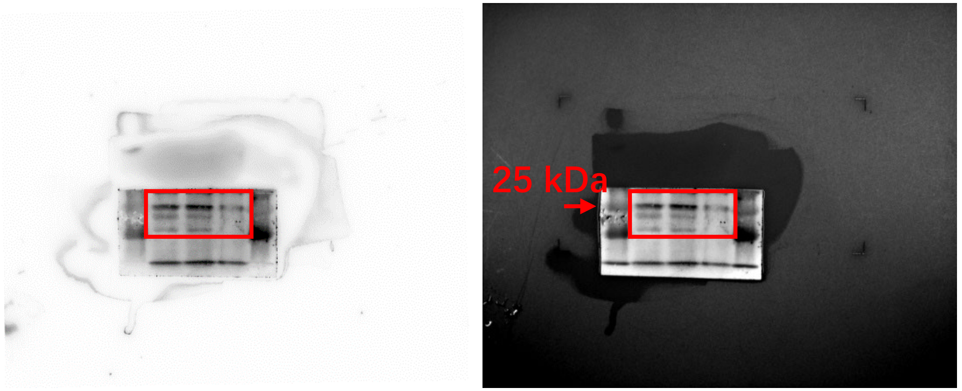

Corresponds to Figure 5C, Anti-β-Tubulin

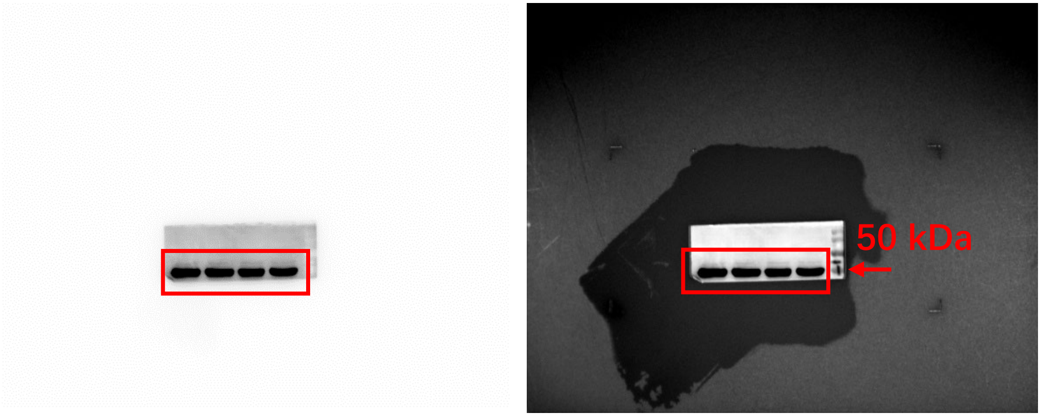

Corresponds to Figure 5D, Anti-VDAC1

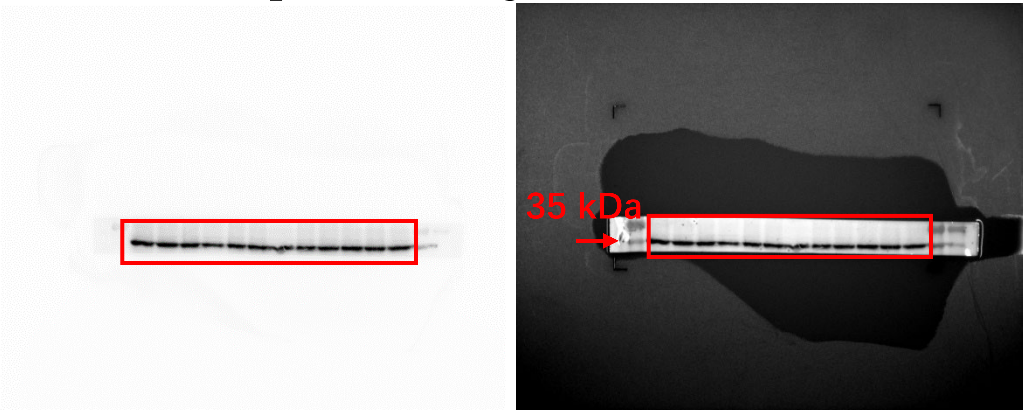

Corresponds to Figure 5E, Anti-β-Tubulin

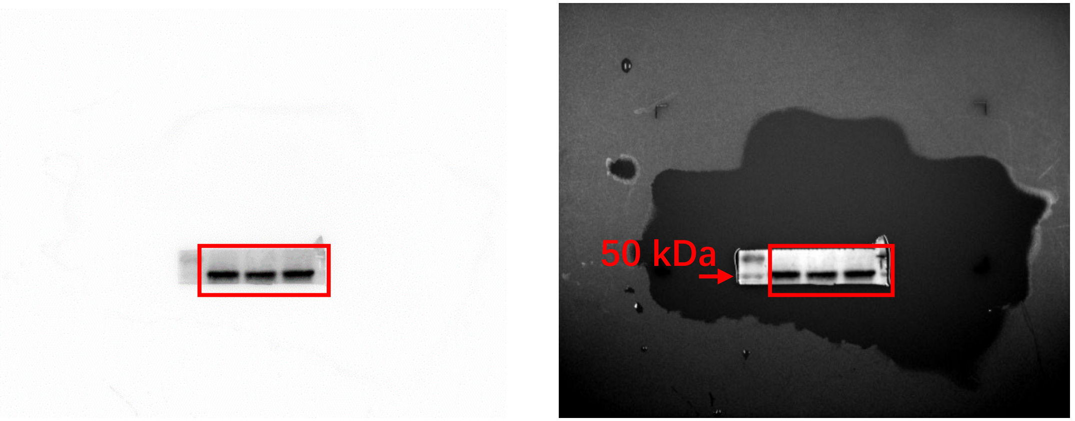

Corresponds to Figure 5E, Anti-Ac-k

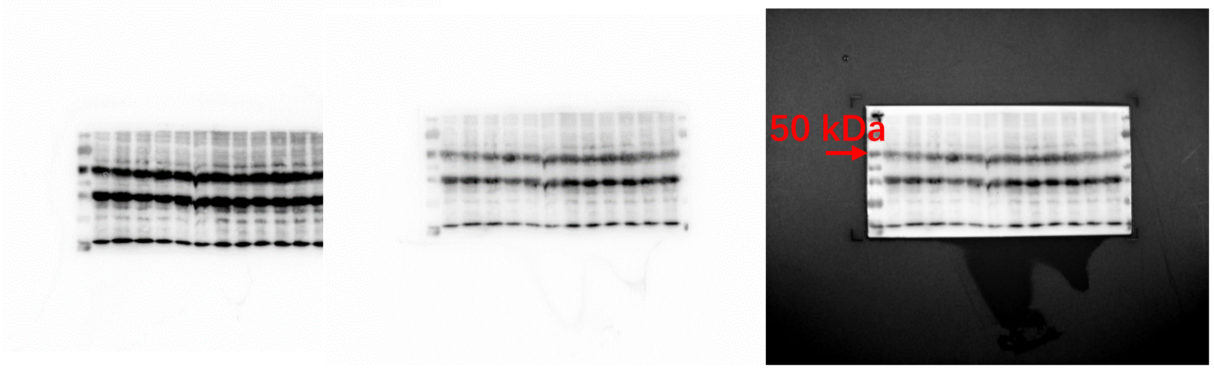

Corresponds to Figure 5E, Anti-VDAC1

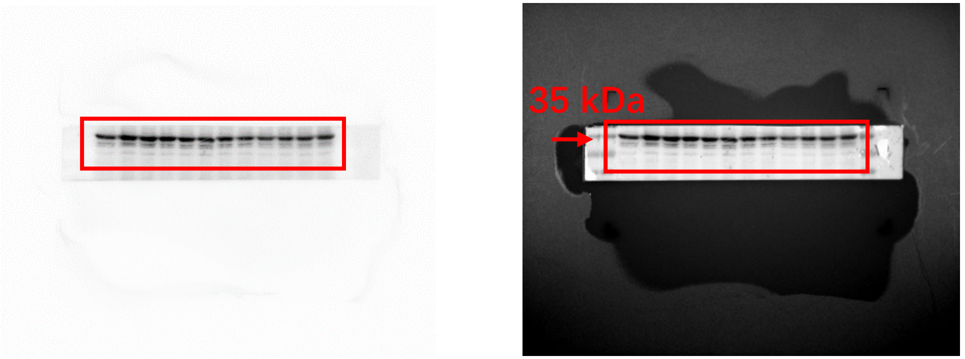

Corresponds to Figure 5F, Anti-P-AMPKα

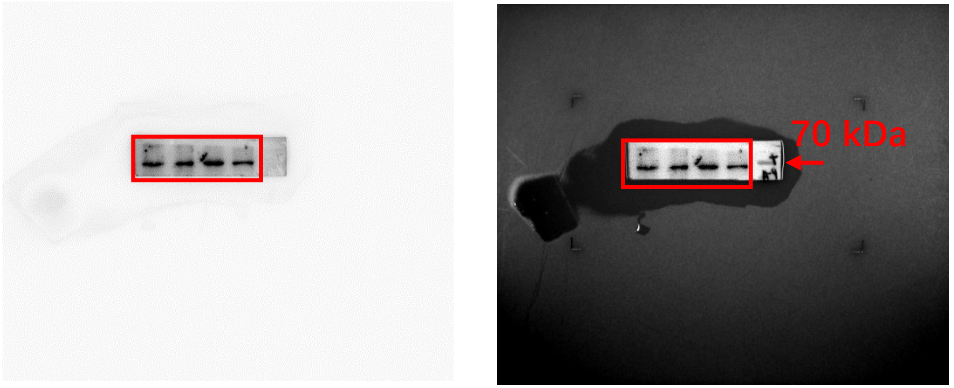

Corresponds to Figure 5F, Anti-AMPKα

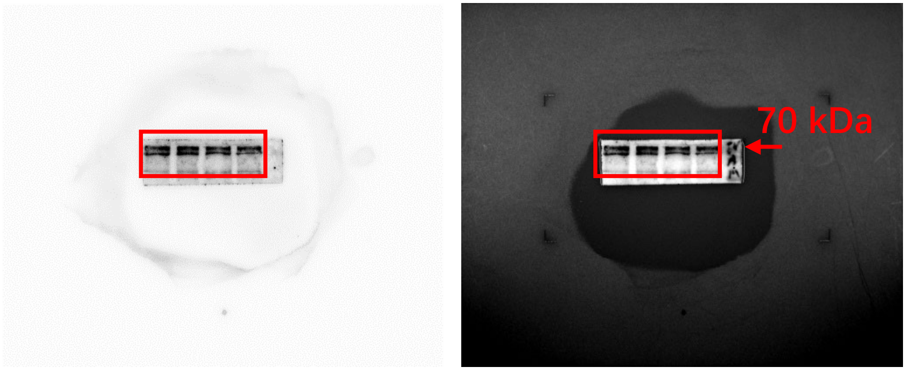

Corresponds to Figure 5F, Anti-P-MFF

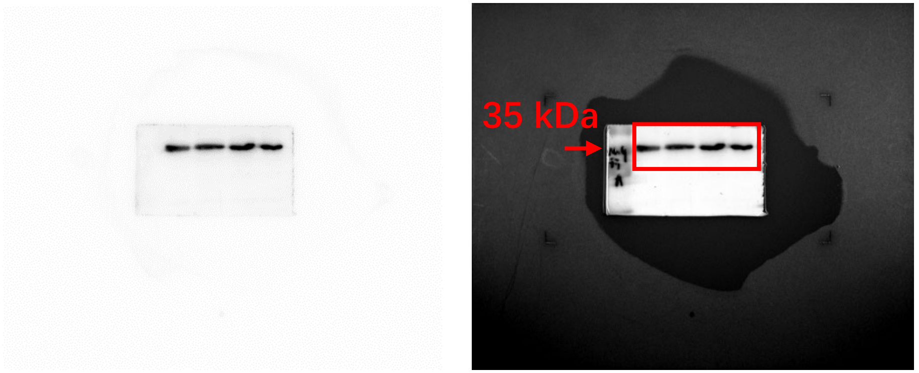

Corresponds to Figure 5F, Anti-MFF

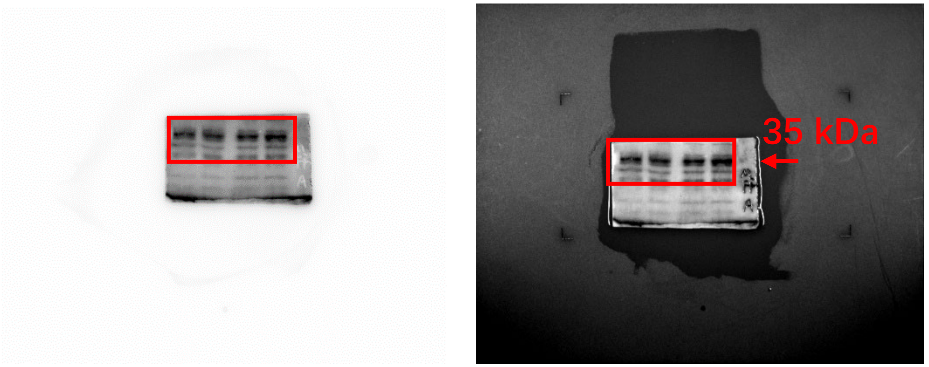

Corresponds to Figure 5F, Anti-GAPDH

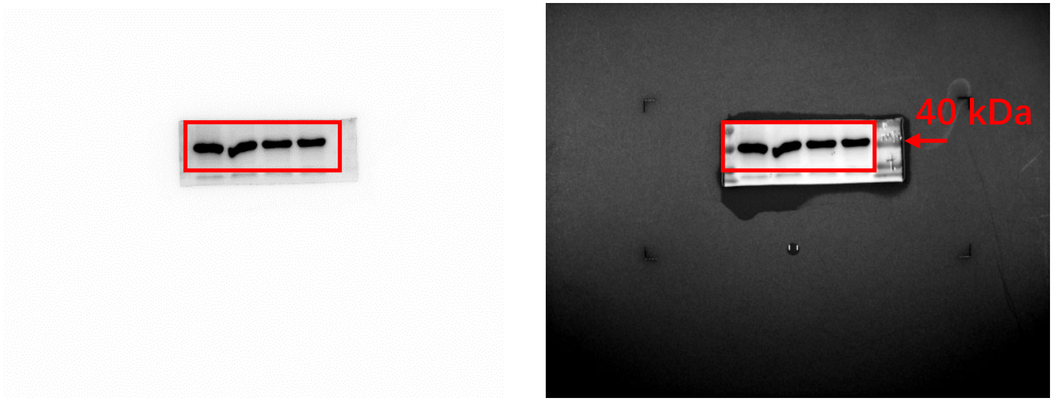

Corresponds to Figure 5G, Anti-β-Tubulin

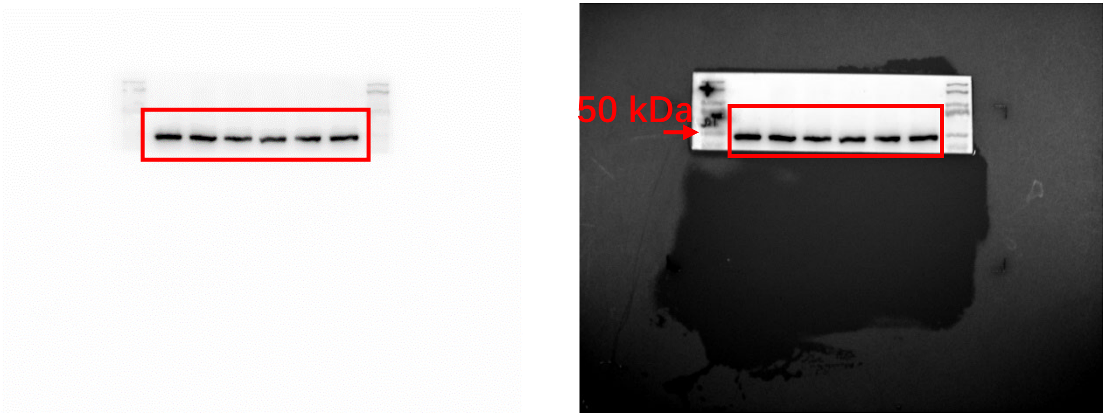

Corresponds to Figure 5G, Anti-P-AMPKα

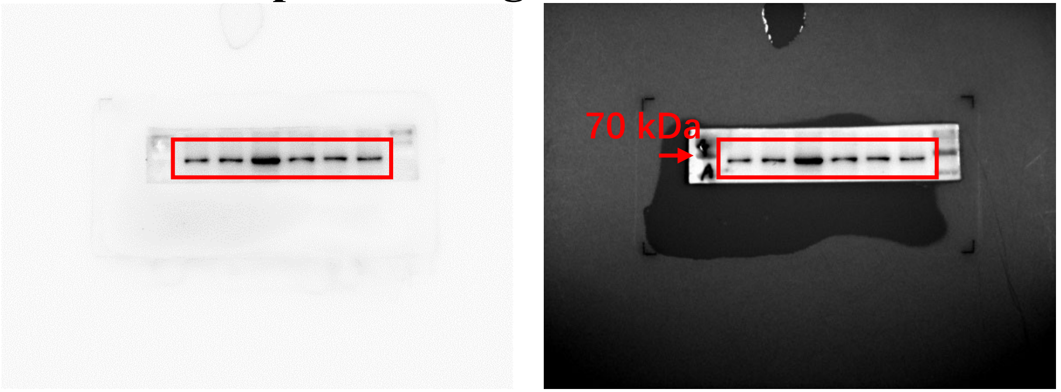

Corresponds to Figure 5G, Anti-AMPKα

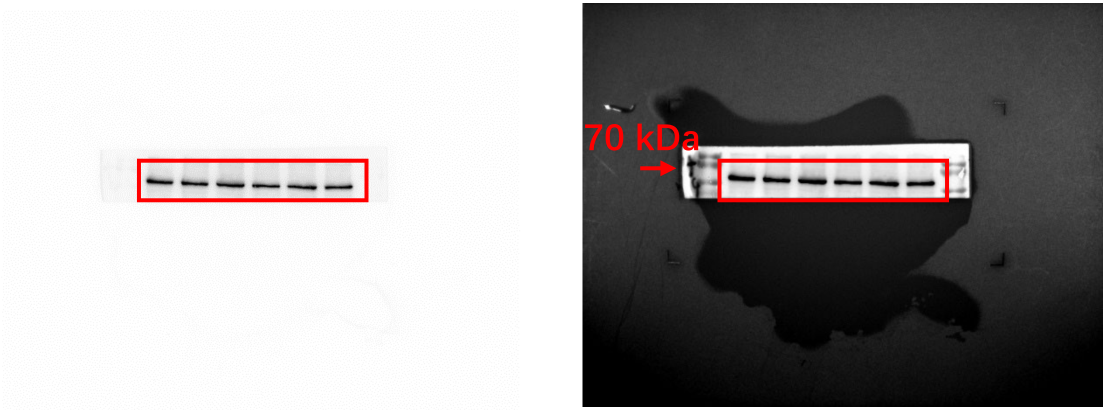

Corresponds to Figure 5G, Anti-P-MFF

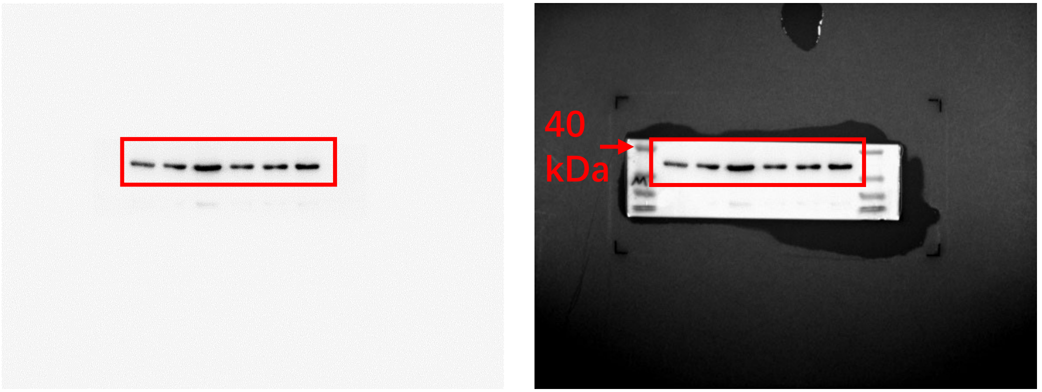

Corresponds to Figure 5G, Anti-MFF

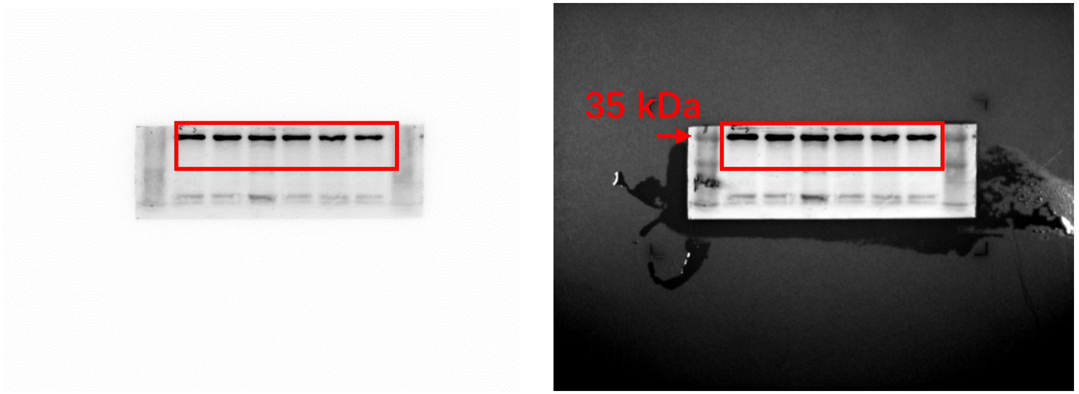

Corresponds to Figure 6C, top panel, Anti-MYC

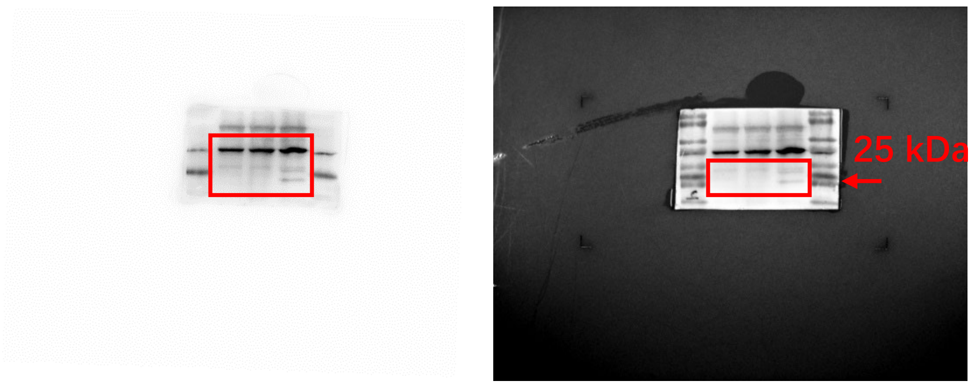

Corresponds to Figure 6C, middle panel, Anti-GFP

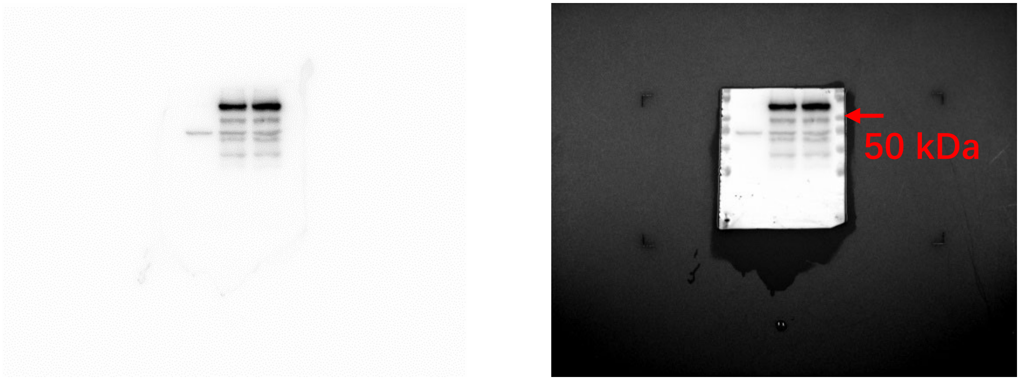

Corresponds to Figure 6C, input, Anti-GFP

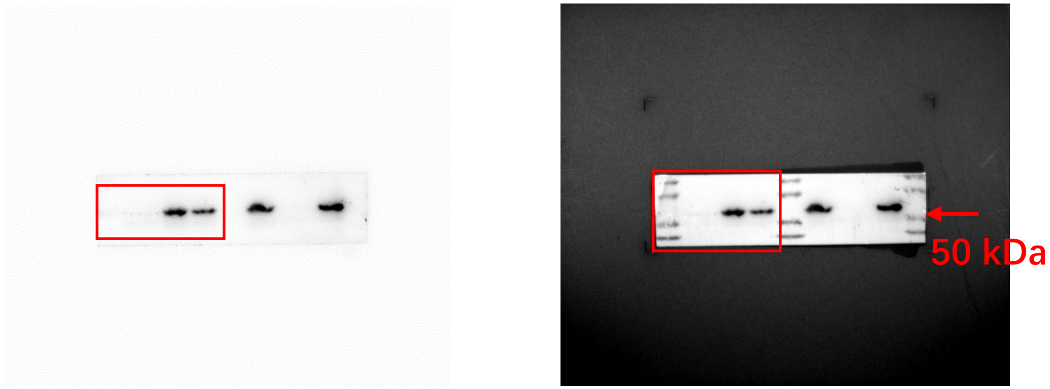

Corresponds to Figure 6C, input, Anti-MYC

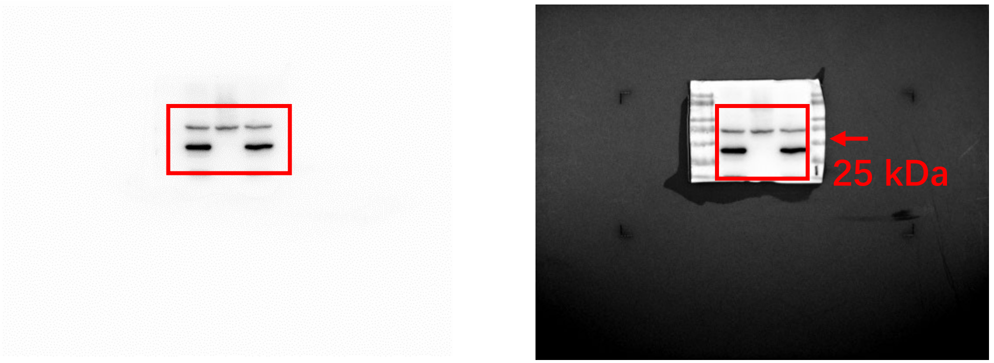

Corresponds to Figure 6H, Anti-Ac-k

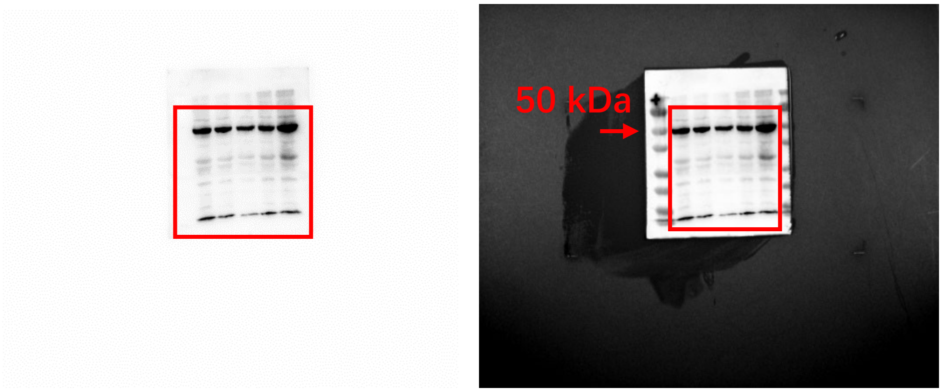

Corresponds to Figure 6H, Anti-VDAC1

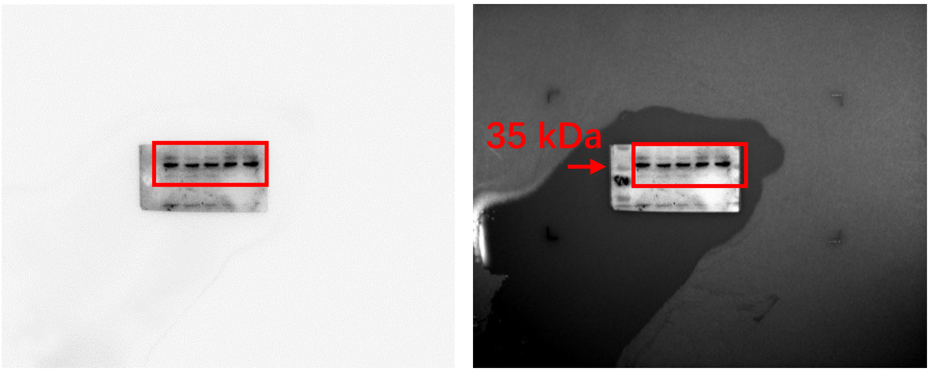

Corresponds to Fig. 2B, Anti-TFAM

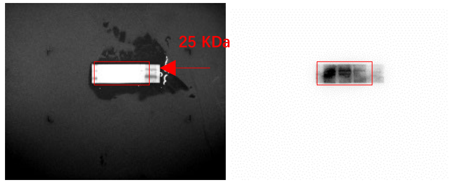

Corresponds to Fig. 4C, Anti-P-Drp1

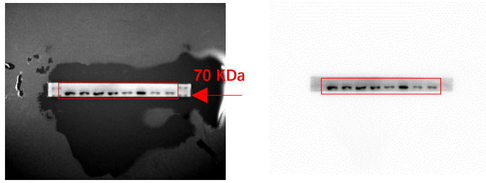

Corresponds to Fig. 2B, Anti-β-Actin

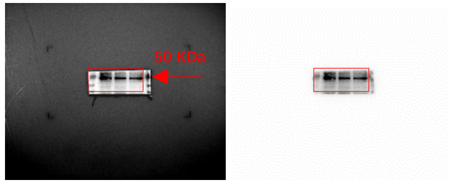

Corresponds to Fig. 4C, Anti-Drp1

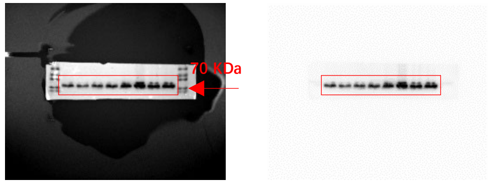

Corresponds to Fig. 4D, Anti-P-MFF

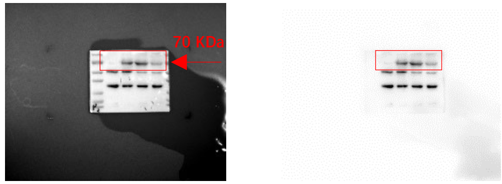

Corresponds to Fig. 4D, Anti-MFF

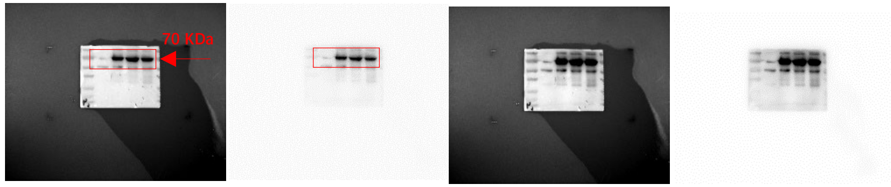

Corresponds to Fig. 4C, Anti-VDAC1

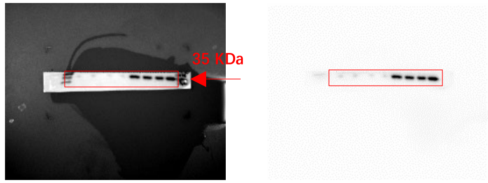

Corresponds to Fig. S5A&B, Anti-P-AMPKα

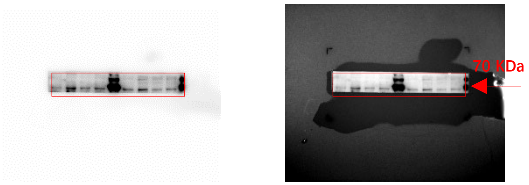

Corresponds to Fig. S5A&B, Anti-Tubulin

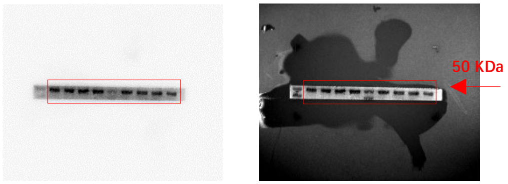

Corresponds to Fig. S5A&B, Anti-TFAM

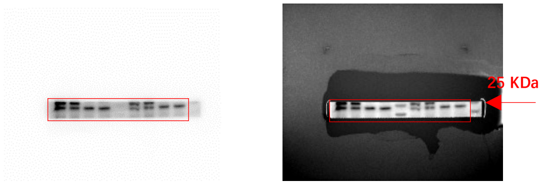

Corresponds to Fig. S5A&B, Anti-P-DRP1

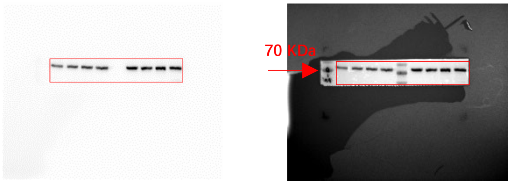

Corresponds to Fig. S5A&B, Anti-Drp1

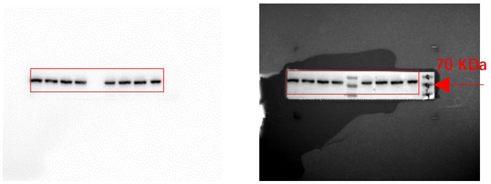

Corresponds to Fig. S5B, Anti-MFF

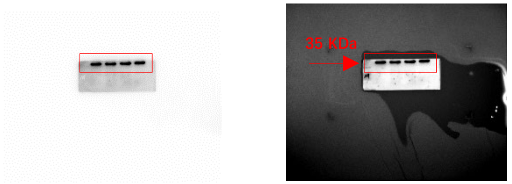

Corresponds to Fig. S5A&B, Anti-AMPKα

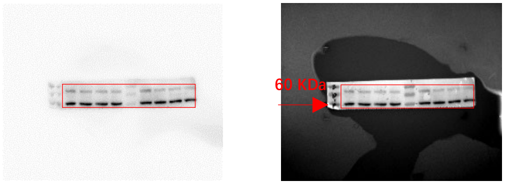

Corresponds to Fig. S5A, Anti-MFF

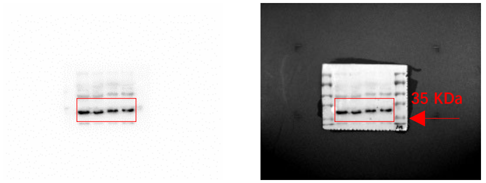

Corresponds to Fig. S5C, Anti-VDAC1

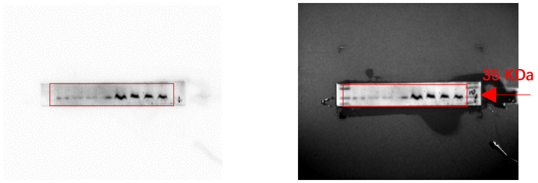

Corresponds to Fig. S5A, Anti-P-MFF

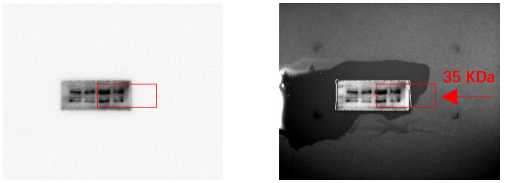

Corresponds to Fig. S5B, Anti-P-MFF

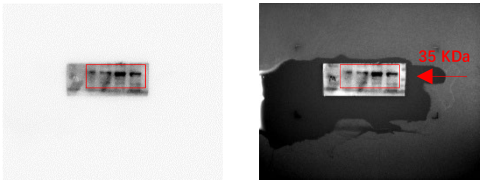

Corresponds to Fig. S5C, Anti-P-Drp1

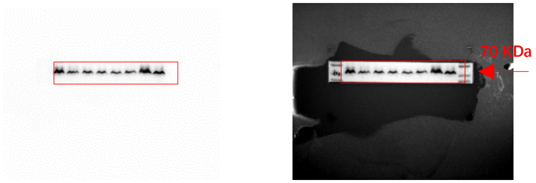

Corresponds to Fig. S5C, Anti-Drp1

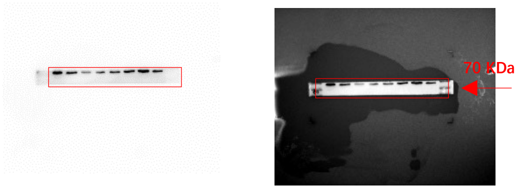

Corresponds to Fig. S5D, Anti-Drp1

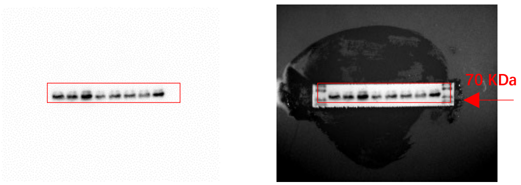

Corresponds to Fig. S5D, Anti-VDAC1

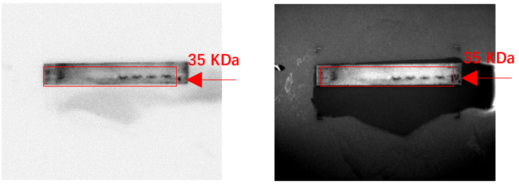

Corresponds to Fig. S5D, Anti-P-Drp1

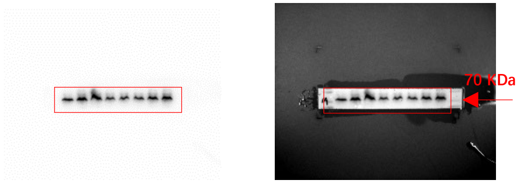

Corresponds to Fig. S6C, Anti-Ac-k

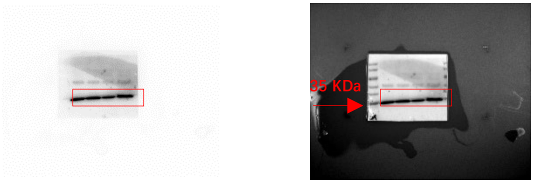

Corresponds to Fig. S6C, Anti-β-Tubulin

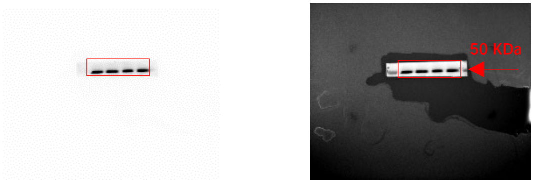

Corresponds to Fig. S6E, Anti-Ac-k

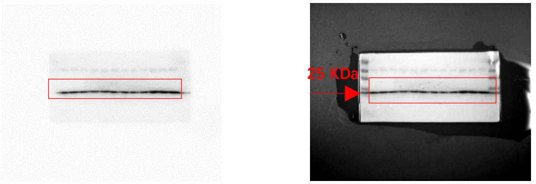

Corresponds to Fig. S6E, Anti-VDAC1

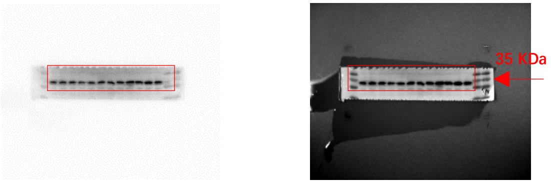

Corresponds to Fig. S6D, Anti-Ac-k

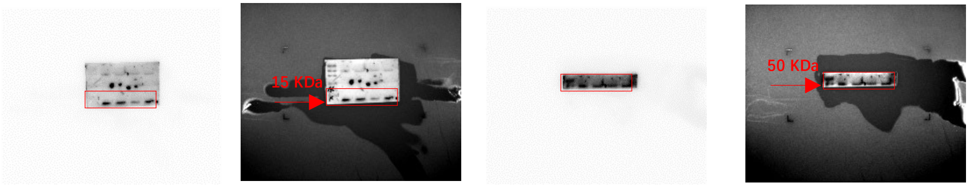

Corresponds to Fig. S6D, Anti-β-Tubulin

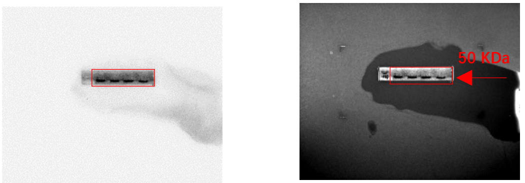

Corresponds to Fig. S6F, Anti-Ac-k

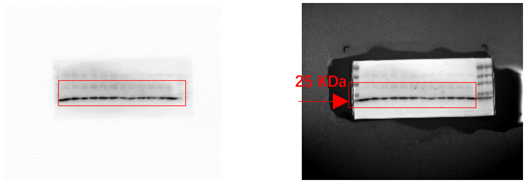

Corresponds to Fig. S6F, Anti-VDAC1

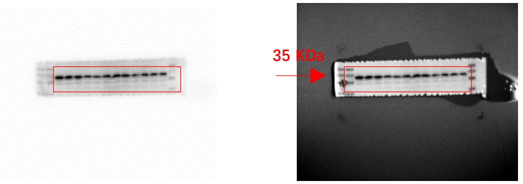

Corresponds to Fig. S6G, Anti-DRP1

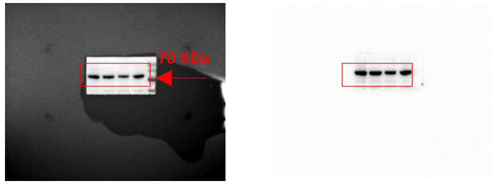

Corresponds to Fig. S6G, Anti-P-Drp1

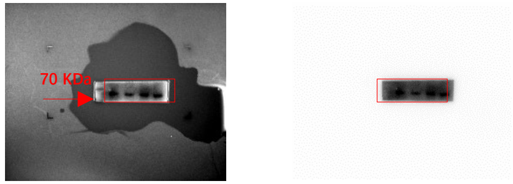

Corresponds to Fig. S6H, Anti-P-Drp1

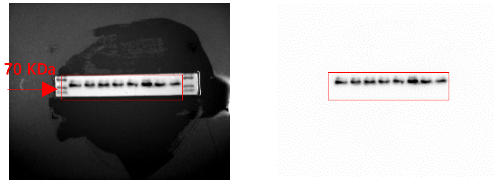

Corresponds to Fig. S6G , Anti-β-Tubulin

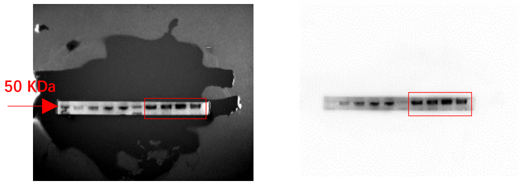

Corresponds to Fig. S6G, Anti-P-AMPKα

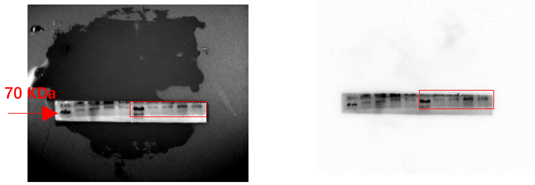

Corresponds to Fig. S6H, Anti-Drp1

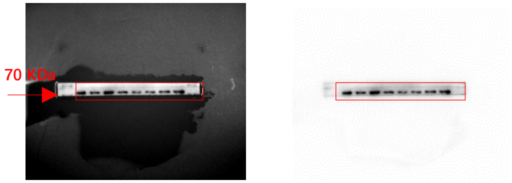

Corresponds to Fig. S6G, Anti-MYC

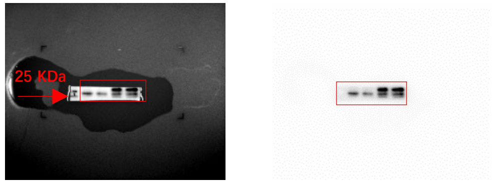

Corresponds to Fig. S6G, Anti-P-MFF

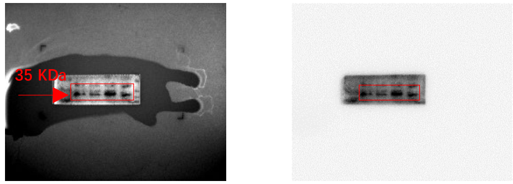

Corresponds to Fig. S6H, Anti-VDAC1

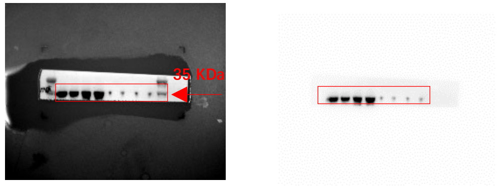

Corresponds to Fig. S6G, Anti-MFF

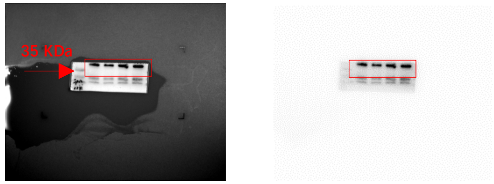

Corresponds to Fig. S6G, Anti-AMPKα

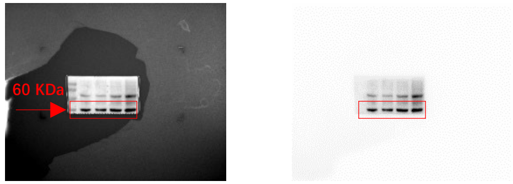

Corresponds to Fig. S6I , Anti-β-Tubulin

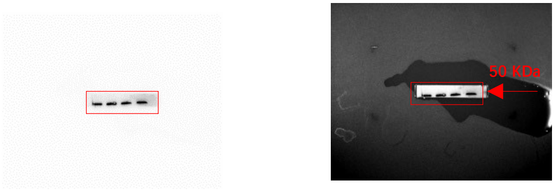

Corresponds to Fig. S6I , Anti-AMPKα

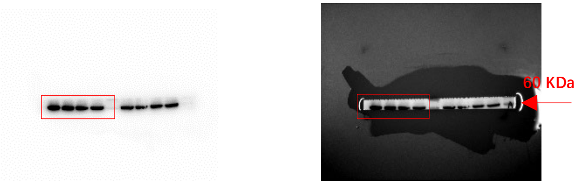

Corresponds to Fig. S6J, Anti-P-Drp1

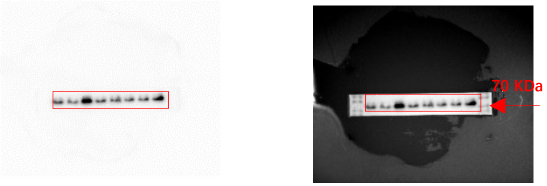

Corresponds to Fig. S6I , Anti-P-AMPKα

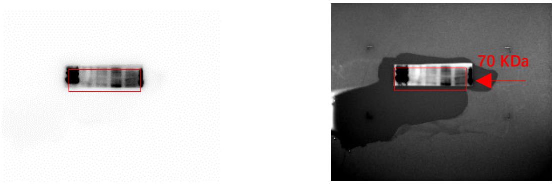

Corresponds to Fig. S6I , Anti-MFF

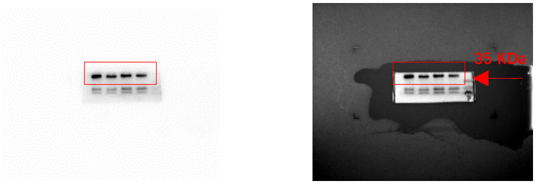

Corresponds to Fig. S6J, Anti-Drp1

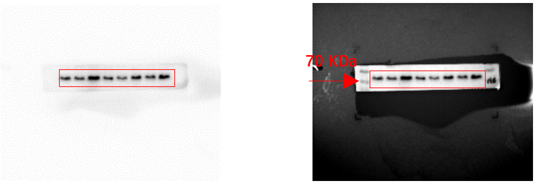

Corresponds to Fig. S6I , Anti-P-DRP1

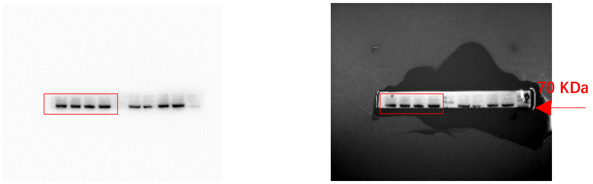

Corresponds to Fig. S6I , Anti-DRP1

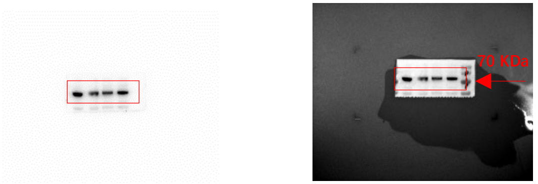

Corresponds to Fig. S6J, Anti-VDAC1

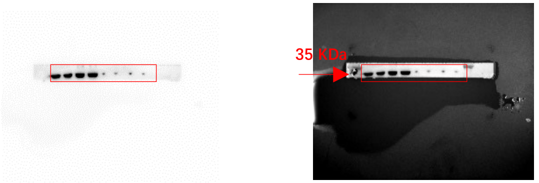

Corresponds to Fig. S6I , Anti-P-MFF

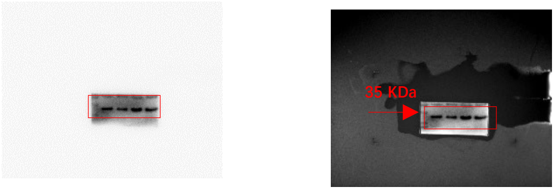

Corresponds to Fig. S6I , Anti-TFAM

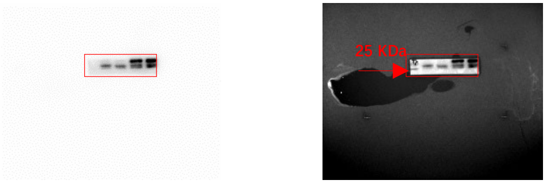

Corresponds to Fig. S6K , Anti-P-MFF

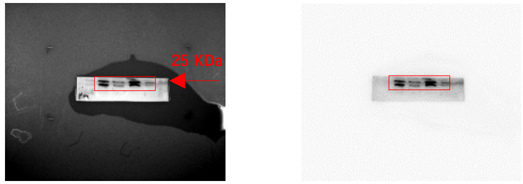

Corresponds to Fig. S6K , Anti-AMPKα

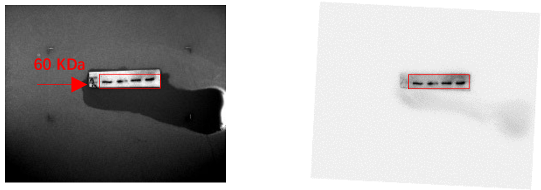

Corresponds to Fig. S6L, Anti-P-Drp1

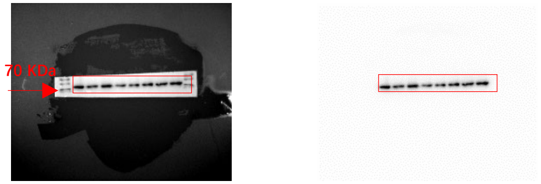

Corresponds to Fig. S6K , Anti-MFF

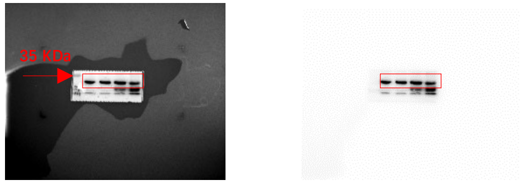

Corresponds to Fig. S6K , Anti-P-AMPKα

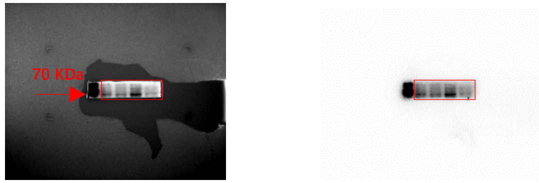

Corresponds to Fig. S6L, Anti-Drp1

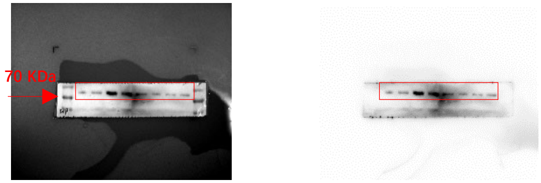

Corresponds to Fig. S6K , Anti-β-Tubulin

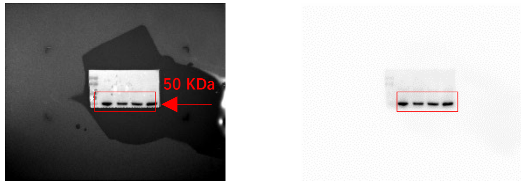

Corresponds to Fig. S6K , Anti-MYC

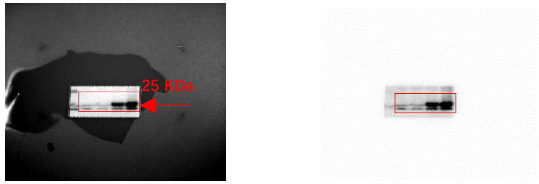

Corresponds to Fig. S6L, Anti-VDAC1

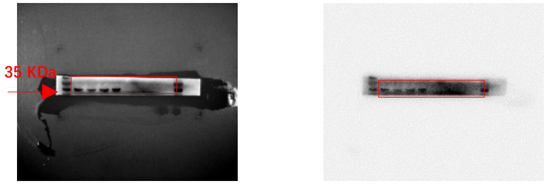

Corresponds to Fig. S7A , Anti-HSP60

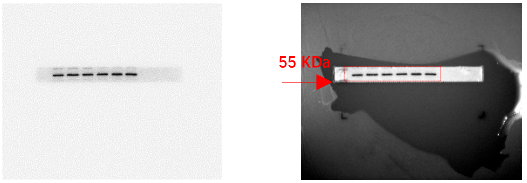

Corresponds to Fig. S7A , Anti-Sirt3

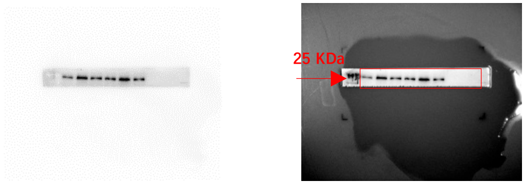

Corresponds to Fig. S7A , Anti-Tom20

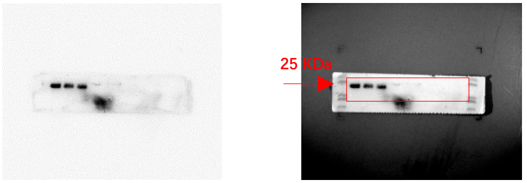

Corresponds to Fig. S7B , Anti-Sirt3

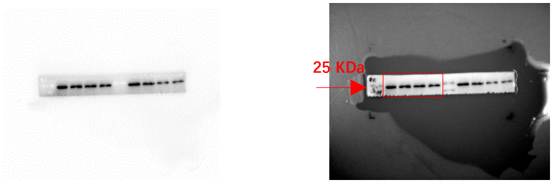

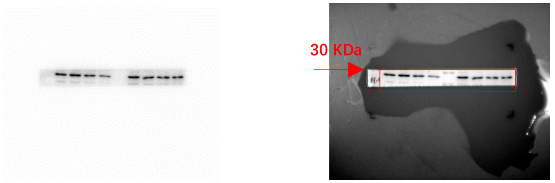

Corresponds to Fig. S7B , Anti-Tom20

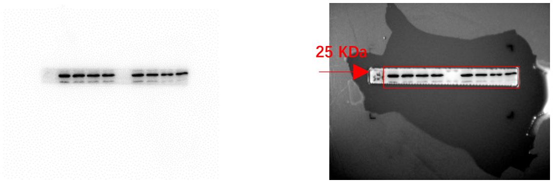

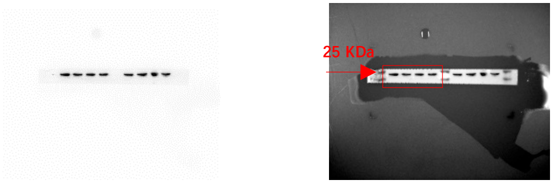

Corresponds to Fig. S7C , Anti-Ac-SOD2

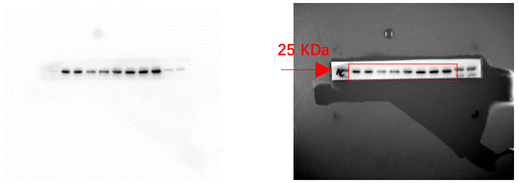

Corresponds to Fig. S7C , Anti-SOD2

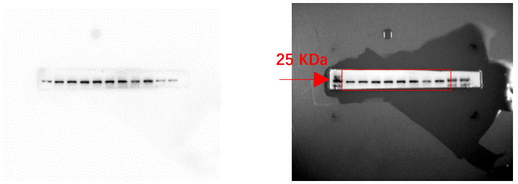

Corresponds to Fig. S7C , Anti-Tom20

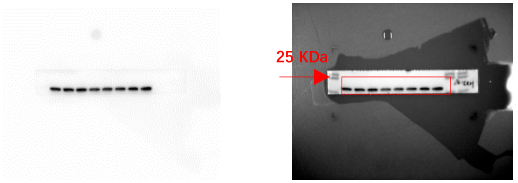

Corresponds to Fig. S8 , Anti-TFAM

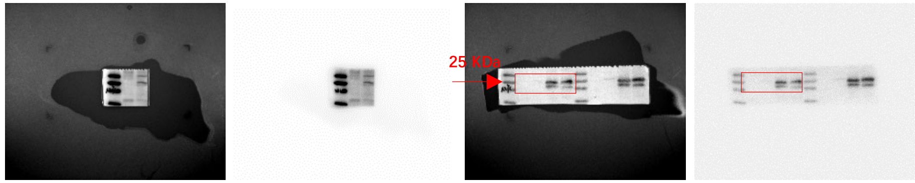

Corresponds to Fig. S8 , Anti-MYC (TFAM) Input

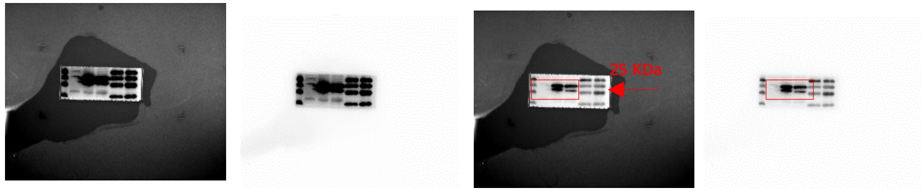

Corresponds to Fig. S8 , Anti-Sirt3

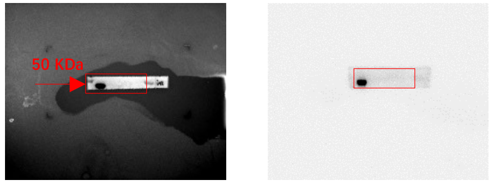

Corresponds to Fig. S8 , Anti-MYC (IP)

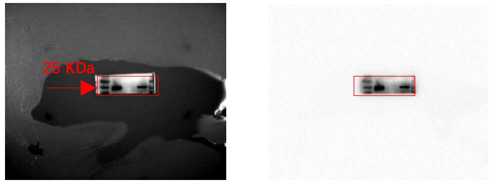

Corresponds to Fig. S8 , Anti-GFP (Sirt3) (IP)/
Corresponds to Fig. S8 , Anti-GFP (Sirt3) (Input)

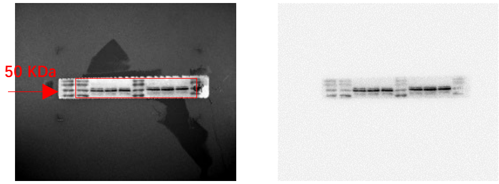

Supplement: Supplementary file 2 — Uncropped_Blots [file 41419_2026_8750_MOESM2_ESM.pdf]
